# Analysis of the Heterogeneity of CD4^+^CD25^+^ T Cell TCR *β* CDR3 Repertoires in Breast Tumor Tissues, Lung Metastatic Tissues, and Spleens from 4T1 Tumor-Bearing BALB/c Mice

**DOI:** 10.1155/2020/3184190

**Published:** 2020-09-24

**Authors:** Teng Zhang, Fangfang Duan, Danhua Su, Long Ma, Jiezuan Yang, Bin Shi, Xiaoyan He, Rui Ma, Suhong Sun, Xinsheng Yao

**Affiliations:** ^1^Department of Breast Surgery, The Affiliated Hospital of Zunyi Medical University, Zunyi, Guizhou, China 563000; ^2^Department of Immunology, Research Center for Medicine & Biology, Innovation & Practice Base for Graduate Students Education, Zunyi Medical University, Zunyi, Guizhou, China 563000; ^3^Department of Infectious Diseases, First Affiliated Hospital, School of Medicine, Zhejiang University, Hangzhou, Zhejiang, China 310003; ^4^Department of Laboratory Medicine, Zunyi Medical University, Zunyi, Guizhou, China 563000

## Abstract

To study the homogeneity and heterogeneity of CD4^+^CD25^+^ T cells receptor *β*-chain complementarity determining region 3 (TCR *β* CDR3) repertoires in breast tumor tissues, lung metastatic tissues, and spleens from 4T1 tumor-bearing BALB/c mice. We used high-throughput sequencing to analyze the characteristics and changes of CD4^+^CD25^+^ TCR *β* CDR3 repertoires among tumor tissues, lung metastatic tissues, and spleens. The diversity of the CD4^+^CD25^+^ TCR *β* CDR3 repertoires in breast tumor tissue was similar to that of lung metastatic tissues and less pronounced than that of spleen tissues. Breast tumor tissues and lung metastatic tissues had a greater number of high-frequency CDR3 sequences and intermediate-frequency CDR3 sequences than those of spleens. The proportion of unique productive CDR3 sequences in breast tumor tissues and lung metastatic tissues was significantly greater than that in the spleens. The diversity and frequency of the CDR3 repertoires remained homogeneous in breast tumors and lung metastatic tissues and showed great heterogeneity in the spleens, which suggested that the breast tissues and lung metastatic tissues have characteristics of CD4^+^CD25^+^ T cells that relate to the tumor microenvironment. However, the number and characteristics of overlapping CDR3 sequences suggested that there were some different CD4^+^CD25^+^ T cells in tumors and in the circulatory immune system. The study may be used to further explore the characteristics of the CDR3 repertoires and determine the source of the CD4^+^CD25^+^ T cells in the breast cancer microenvironment.

## 1. Introduction

Given the link between inflammation and cancer, and the presence of immune cells in the tumor microenvironment, it is very important to study the antitumor immune response and tumor immune escape. CD4^+^CD25^+^T cells is a subset of T cells, which plays an important role in the body inflammation and immune response [1, 2]. Cells surface markers for CD4^+^CD25^+^ also include a small part activated T cells [3, 4]. However, in mice, the identification of Tregs as CD4^+^CD25^+^ T cells has been used widely, representing approximately 5-10% of CD4^+^ T cells in the periphery [5]. Moreover, CD4^+^FoxP3^+^CD25^+^ T cells account for 66.7% of CD25^+^CD4^+^ T cells in mouse spleen [6]. In recent years, research has showed that the absolute number or subset of CD4^+^CD25^+^Tregs increases in peripheral blood, lymph node, and tumor tissue of patients with breast cancer and closely related to the pathological type of breast cancer and progress [4–9]. The number of Tregs in peripheral blood of breast cancer patients with stages III and IV is significantly higher than stages I and II, and cancer tissues are higher than that of adjacent tissues [10]. Metastatic lymph nodes and nonmetastatic lymph nodes in breast cancer patients have different proportions of Tregs and helper T cells (Th1, Th2, Th17, and Tfh cells) [11]. The number of Tregs in advanced breast cancer patients also increased significantly in lymph node lung metastasis tissues, suggesting that it may promote lymph node metastasis of tumor cells in breast cancer microenvironment [12]. In the tumor microenvironment of primary breast cancer and lymph node metastasis, the increased proportion of Tregs is associated with the high expression of indoleamine 2 and 3-dioxygenase (IDO) [13], but is the very tumor site tissue of Th17 cells related to IL-17A, RORC, and CCR6 upregulation [14], and breast cancer patients with mononuclear dendritic cells mediate allogeneic CD3^+^CD2^−^Foxp3-T cells into CD25^+^T cells [15]. At the same time, Gökmen-Polar et al. found that there was no significant difference between the Foxp3^+^Tregs in different pathological types of breast cancer patients with lymph node metastasis and nonmetastases. This suggests the complexity of Tregs in the breast cancer microenvironment [16]. The origin, differentiation, activation mechanism, and characteristics of Tregs in breast cancer have not yet been clarified.

In recent years, with the development of HTS (high-throughput sequencing, by measuring billions of short DNA or RNA fragments) monitoring TCR CDR3 repertoires [17] and its potential application in tumor research [18, 19], the homogeneity and heterogeneity of CDR3 repertoires between different parts of breast tumor tissues and adjacent tissues of breast tumor tissues, or TIL cells and peripheral blood T cells were studied in several laboratories. HTS has been widely used to analyze the TIL cells CDR3 repertoires of colorectal cancer [20], ovarian cancer [21], pancreatic cancer [22], esophageal cancer, gastric cancer [23], brain glioma [24], and multiple human tumors [25]. However, there is still a lack of CD4^+^CD25^+^ T cells TCR *β* CDR3 repertoires between tumor tissue and the circulatory immune system. We found that there were some differences in the TIL cell CDR3 spectratyping between breast cancer tissues and peripheral blood [26, 27]. In this study, 4T1 breast cancer cells were transplanted into BALB/c mice, and the homogeneity and heterogeneity of CD4^+^CD25^+^ T cells TCR *β* CDR3 repertoires in breast tumor tissues, lung metastatic tissues, and spleens were monitored by the HTS technique. These data may be used to further explore the CDR3 repertoires characteristics and source of the breast cancer microenvironment of CD4^+^CD25^+^ T cells. Raw data can be accessed in https://data.mendeley.com/datasets/jn7pt7y74d/draft?a=437968bf-1bc5-4061-bd75-3a5c65b8157e.

## 2. Materials and Methods

### 2.1. Materials

Mouse mammary carcinoma cells (4T1 cells) were acquired from the Shanghai Institutes for Biological Sciences. BALB/c female mice, 4-week-old, were purchased from Experimental Animal Ltd., Hospital of Third Military Medical University, and were fed in the SPF-grade facility of Zunyi Medical College Experimental Animal Center. The ethics statement and the animal experiments were performed in accordance with the guidelines of the Animal Care and Use of Laboratory Animals (Ministry of Health, China, 1998). The experimental procedures were approved by the ethical guidelines of Zunyi Medical College Laboratory Animal and Use Committee. The following reagents and instruments were used: CD4^+^CD25^+^ T cells Isolation Kit, CD4-PC5.5, CD3*ε*-FITC, and CD25-PE antibody (mouse, Miltenyi Biotec, Germany); Fatal bovine serum (FBS), trypsin, RPMI-1640 medium (Gibco, USA); DNA QIAamp DNA Mini Kit, Dneasy® Blood & Tissue Kit (Qiagen, Germany); Ficoll Lymphocyte Separation Solution (Beijing Solaibao Technology Co., Ltd.); and Gentle MACS dissociator (Miltenyi Biotec, Germany).

### 2.2. Methods

#### 2.2.1. Tumor-Bearing Mice Model (4T1 Cells)

Mouse mammary carcinoma cells (4T1 cells) were maintained in RPMI-1640 medium supplemented with 10% heat-inactivated fetal calf serum (FCS); cells were detached with 0.2% trypsin-EDTA, washed three times with PBS, and resuspended in PBS at 1 × 10^7^/ml. One hundred microliters of cell suspension (1 × 10^6^ cells) was injected into mammary fat pads of female BALB/c mice. Three mice were euthanized at 8 weeks old, and the lungs, tumors, and spleens were isolated. The tumors were formalin-fixed and paraffin-embedded with H&E for histology analysis, note: B=breast tumor tissue, L=lung metastatic tissue, and S=spleen.

#### 2.2.2. Isolation of CD4^+^CD25^+^Cells with MACS

Breast tumor tissues, lung metastatic tissues, and spleens of tumor-bearing mice were separated, and single-cell suspensions of each tissue sample were obtained using the Gentle MACS dissociator. For MACS separation, the CD4^+^CD25^+^ T cells Isolation Kit was used, and CD4^+^CD25^+^ T cells were isolated according to the manufacturer's instructions. CD4^+^CD25^+^T cells in 1 × 10^5^ cells/200 *μ*l were incubated on ice for 30 min with anti-CD4-PC5.5, anti-CD3*ε*-FITC, and anti-CD25-FE. Cells were acquired on a flow cytometer (Beckman Coulter Gallios).

#### 2.2.3. HTS of the CD4^+^CD25^+^ TCR *β* CDR3 Repertoires

DNA was extracted from CD4^+^CD25^+^ T cells with the DNeasy DNA Mini Kit according to the manufacturer's instructions. Then, DNA was identified using 1% agarose gel electrophoresis. DNA was stored in QIA safe DNA tubes (Qiagen) and was sent to the USA Adaptive Biotechnologies company for Immuno SEQ, the company responsible for the completion of each sample concentration and purity testing, sample quality control, and HTS of the mouse TCR *β* CDR3 repertoires (Illumina HTS) [28, 29].

#### 2.2.4. Analysis of the Composition and Characteristics of the CDR3 Repertoires

Immuno SEQ and IMGT/High V-QUEST Systems (http://www.imgt.org) were used to analyze the composition and characteristics of the CDR3-repertoire sequences in each sample. (1) Out-of-frame and unresolved sequences were screened out of the CDR3 repertoires used for HTS sequencing. The total productive CDR3 sequences were obtained, and the unique productive CDR3 sequences were calculated. (2) The screened total productive and unique productive CDR3 sequences were used to analyze CDR3 diversity (clone level), 1/Ds = 1/[1 − sigma(Ni (Ni − 1))/(N(N − 1))], and the larger the value of 1/DS, the larger the sample diversity. (3) The distribution of AA within the CDR3 repertoires of high frequency (greater than 0.05%), intermediate frequency (0.01-0.05%), low frequency (less than 0.01%), the clone value-added ratio, composition and characteristics of the top five/ten/twenty highest-frequency sequences in CDR3 repertoires, the usage and gene recombination of TRBV-TRBJ, and the length of CDR3 sequences repertoires were analyzed here. The number and rate of overlap among CDR3 sequences was analyzed using Venn diagram software. The experimental data of three kinds of different tissue parts were compared by the average of statistics, one-way ANOVA, chi-squared tests, and other statistical methods. *p* < 0.05 was considered to be statistically significant. All statistically significant differences are indicated as ^∗^*p* < 0.05, ^∗∗^*p* < 0.01, and ^∗∗∗^*p* < 0.001 [30–32].

## 3. Results

### 3.1. 4T1 Tumor-Bearing Mice Model Establishment and Sample Collection

**Figure 1:**
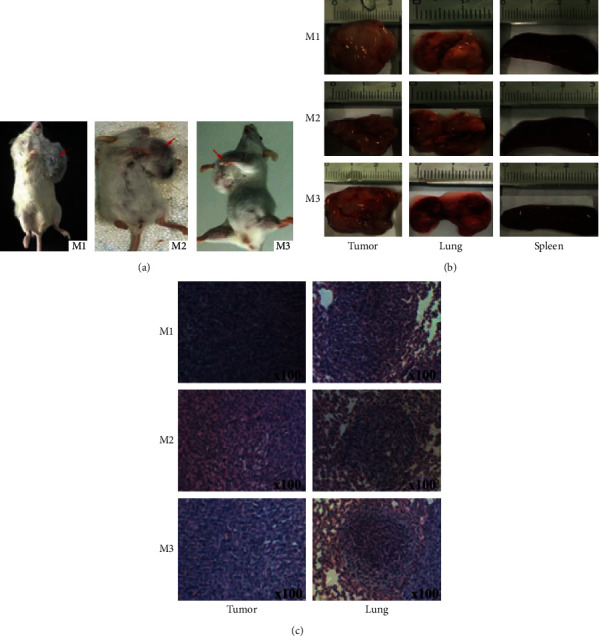
Three 4T1 tumor-bearing BALB/c mice (a) and the collection of samples of breast tumor tissues, metastatic lung tissues, and spleens (b) and pathological sections of HE staining of breast tumor tissues and lung metastatic tissues (c).

The results of mammary tumor cells line of 4T1 cells (10^6^/mouse) within the mammary fat pad showed that the percentage of tumor incidence reached 100%. Three mice (Figure 1(a)) were euthanized at 8 weeks old, and the lungs, tumors, and spleens (Figure 1(b)) were isolated. The tumors were formalin-fixed and paraffin-embedded with H&E for histology analysis (Figure 1(c)).

### 3.2. Isolation and Identification of CD4^+^CD25^+^ T Cells

**Figure 2:**
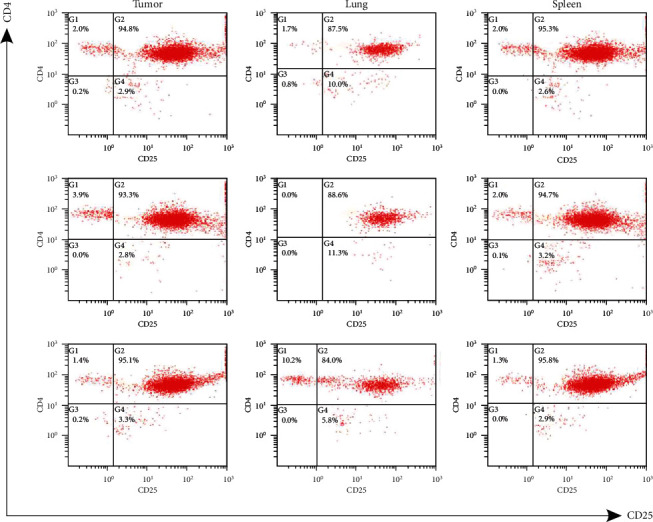
FCM detection of breast tumor tissues, lung metastatic tissues, and spleens sorted CD4^+^CD25^+^ T cells in three 4T1 tumor-bearing BALB/c mice.

CD4^+^CD25^+^ T cells of FCM detection were all above 84% (Figure 2); the DNA concentration and purity of CD4^+^CD25^+^ T cells were shown in Figure S1, Supplement Table 1, and Supplement Tables 2.

### 3.3. The Characteristics of CD4^+^CD25^+^ T cells CDR3 Repertoires

Total and unique productive TCR *β* CDR3 sequences of CD4^+^CD25^+^ T cells in nine tissue sections from three 4T1 tumor-bearing BALB/c mice were shown in Supplement Table 2 and Supplement Table 3, which accorded with the quantity of CDR3 repertoire comparative analysis.

### 3.4. The Diversity of CDR3 Repertoires

**Figure 3:**
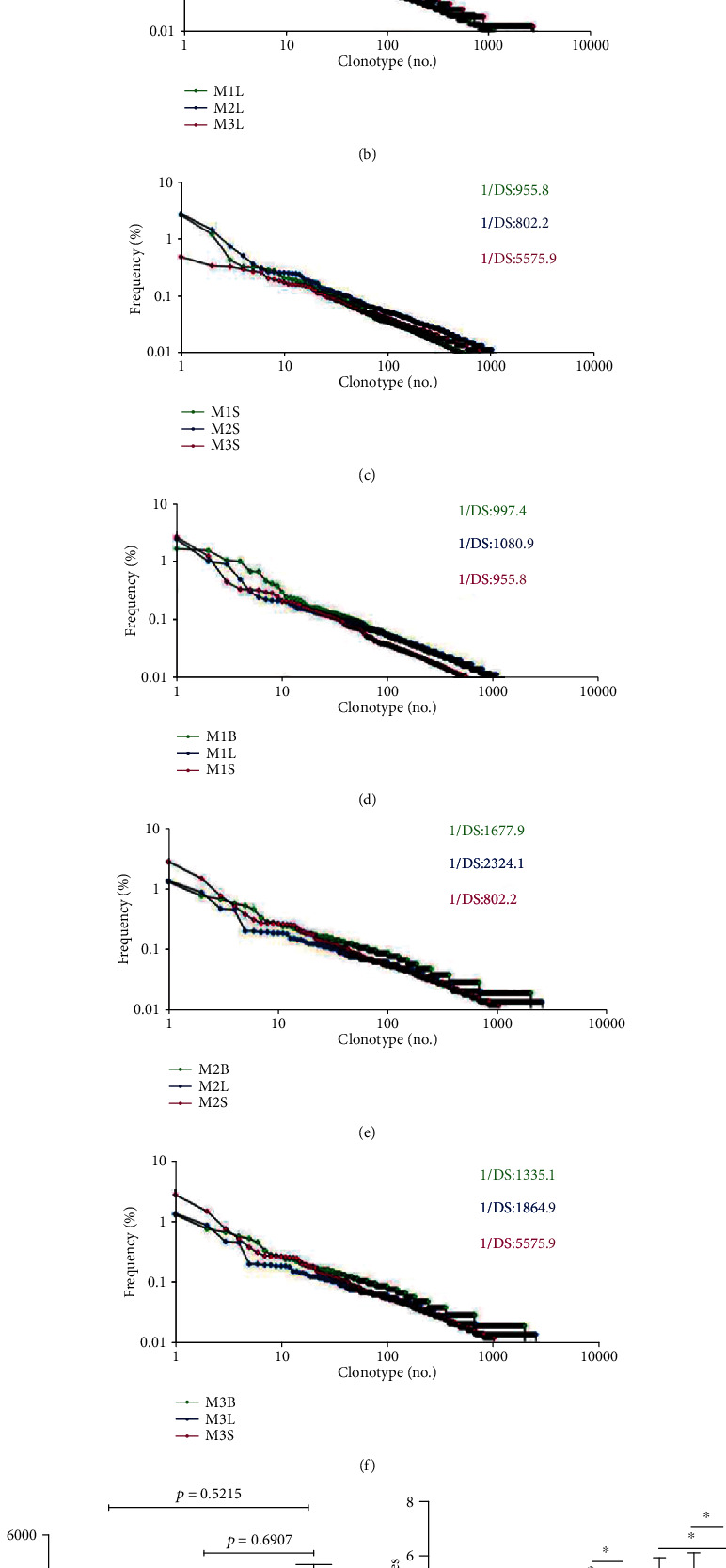
Clonotypes distribution plots (1/DS) (a–f) and statistical analysis of 1/DS (g) of CD4^+^CD25^+^ T cell TCR *β* CDR3 repertoires among tissues from three 4T1 tumor-bearing BALB/c mice; statistical analysis (h) of the five/ten/twenty most highly expanded clones of CDR3 repertoires in three 4T1 tumor-bearing BALB/c mice (*n* = 3,^∗^*p* ≤ 0.05).

The 1/Ds of CDR3 repertoires of CD4^+^CD25^+^ T cells in nine tissues of three mice were shown in Figure 3. The diversity of breast tumor tissues was the smallest, lung metastatic tissues were slightly higher than that of breast tumor tissues, and spleens were the highest, but there was no significant difference among the three tissues (Figure 3(g)).

### 3.5. The Cloning Proliferation of CDR3 Repertoires

CD4^+^CD25^+^ T cells CDR3 repertoires at three tissue distribution of low (less than 0.01%), intermediate (0.01–0.05%), and high (greater than 0.05%) frequency of clonal proliferation (different sequences represent various T cells, and T cells will amplify when they encounter antigens, but different T cells have different amplification frequencies) such as Figure S2. Among them, the distribution of high-frequency and intermediate-frequency was that breast tumor tissues were higher than lung metastatic tissues, and both were greater than spleens. The distribution of low frequency was that spleens were higher than lung metastatic and breast tumor tissues, but no significant statistical difference was found.

Characteristic and proportions of the AA/TRBV/TRBJ with the top five/ten/twenty high-frequency CDR3 sequences in three mice were shown in Tables 1–10. Three of the five high-frequency CDR3 sequences were shared between breast tumor and lung metastatic tissues in Mouse 1 and Mouse 3; in addition, three of the five high frequency CDR3 sequences were shared between breast tumor and spleens in Mouse 3(Table 4). Six of the ten and four of ten high-frequency CDR3 sequences were shared between breast tumor and lung metastatic tissues in Mouse 1 and Mouse 3, respectively; in addition, five of the ten high-frequency CDR3 sequences were shared between breast tumor and spleens in Mouse 3 (Table 4). Eight of the twenty and five of twenty high-frequency CDR3 sequences were shared between breast tumor and lung metastatic tissues in Mouse 1 and Mouse 3, respectively, and five of the twenty, four of the twenty, eight of the twenty high frequency CDR3 sequences were shared between breast tumor and spleens in Mouse 3(Table 4). There was a statistical difference in the overall proportions of the top five/ten/twenty high-frequency CDR3 sequences between breast tumor and spleens and between lung metastatic tissues and spleens. The overall proportions of the top five/ten/twenty highfrequency CDR3 sequences of breast tumor and lung metastatic tissues were higher than those of spleens (Figure 3(h)).

### 3.6. Overlap of CDR3 Repertoires

**Figure 4:**
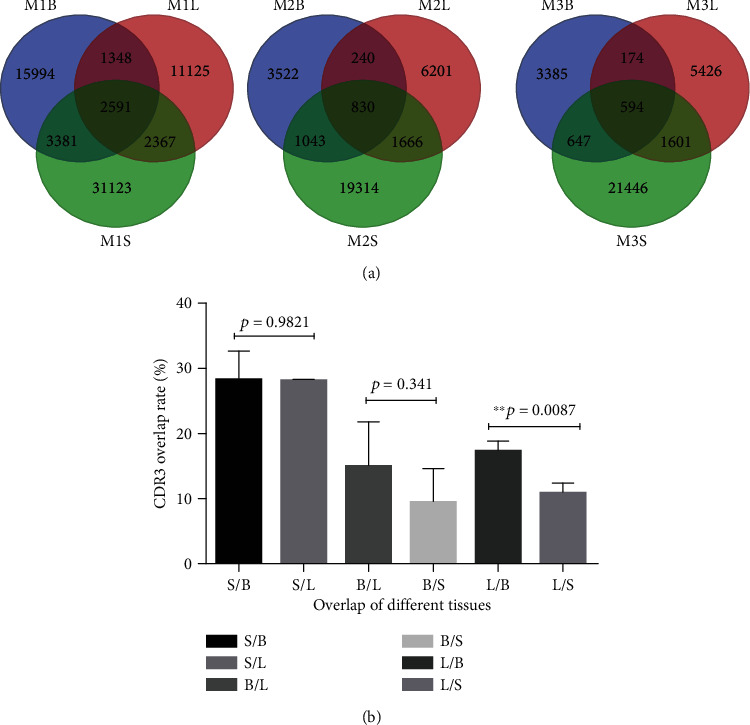
The number of public sequences of CD4^+^CD25^+^ T cells TCR *β* CDR3 repertoires among three tissues of each 4T1 tumor-bearing BALB/c mice (a); the number and ratio (Supplement Table 4-1) and the statistical analysis (b) of public CDR3 sequences from their respective unique productive CDR3 repertoires in each type of tissue (*n* = 3, ^∗∗^*p* < 0.01).

Among the three tissues of each mouse, the overlap unique productive CDR3 sequences are shown in Figure 4(a). The ratio and proportion of public CDR3 sequences in each tissue were found in Supplement Table 4-1. The public CDR3 sequences in spleens were higher than those in breast tumor tissues and lung metastatic tissues. The ratio of public CDR3 sequences in lung metastatic tissues was higher than that of spleens (^∗∗^*p* < 0.01). The proportion of public CDR3 sequences in breast tumor tissues and lung metastatic tissues was higher than that in spleens (Figure 4(b)).

**Figure 5:**
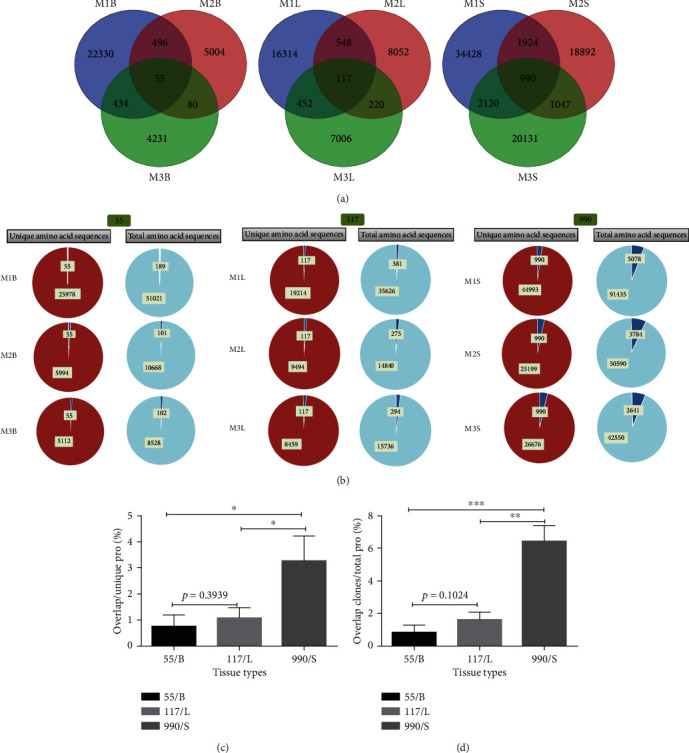
The number of public CDR3 sequences from CD4^+^CD25^+^ T cells TCR *β* CDR3 repertoires among same tissues from three 4T1 tumor-bearing BALB/c mice (a); the number and ratio (b) and the statistical analysis (c) of public CDR3 sequences from their respective unique productive CDR3 repertoires and total productive CDR3 repertoires, respectively.

The number and proportion of public CDR3 sequences in unique productive CDR3 repertoires in the same tissue parts of three mice were shown in Figure 5(a), Supplement Table 4-2. The proportion of public CDR3 sequences in unique productive and total productive CDR3 repertoires was showed in Figure 5(b). The lung metastatic tissues and breast tumor tissues were lower (no difference), but compared with spleens, there were significant differences (Figures 5(c) and 5(d)).

**Figure 6:**
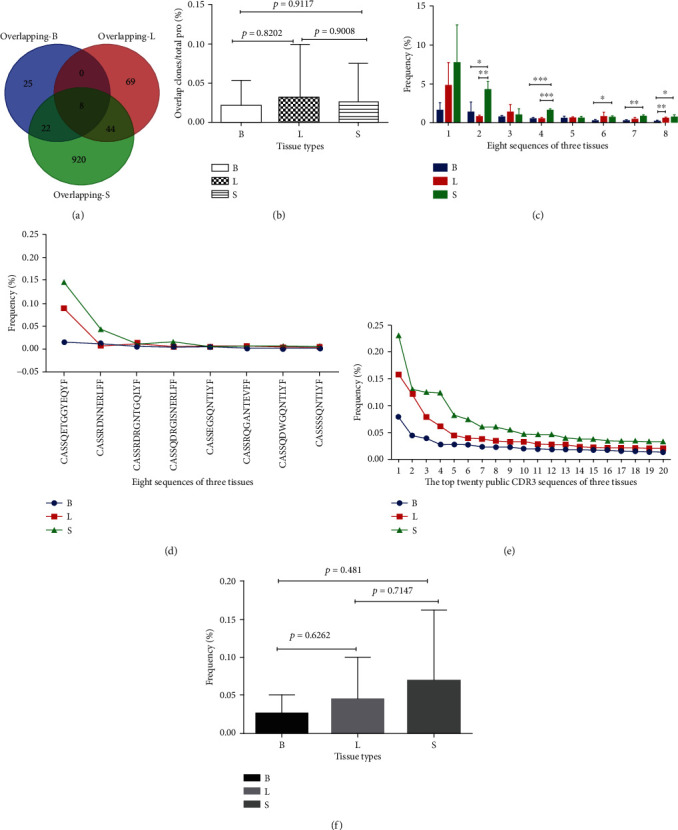
Total overlapped CDR3 sequences among breast tumor tissues, metastatic lung tissues, and spleens from three 4T1 tumor-bearing BALB/c mice (total of nine tissues) (a); statistical analysis comparison of the ratio of cloning proliferation of eight CDR3 sequences overlapping in nine tissues from the CDR3 repertoires: comparison of the three kinds of tissues (b); single comparison (c, d) (Supplement Table 1, ^∗^*p* < 0.05, ^∗∗^*p* < 0.01, ^∗∗∗^*p* < 0.001, *n* = 3); the distribution and proportion statistical analysis of the top 20 sequences overlapping in the three tissues were compared (e, f).

The number of public CDR3 sequences of breast tumor tissues, lung metastatic tissues, and spleens in the three mice was shown Figure 6(a). There was no significant difference in the total proportion of the overlap eight sequences between the three tissues (Figure 6(b)). However, when there were eight sequences, there was a significant difference between the three organizations (Figures 6(c) and 6(d)). The proportion of eight CDR3 AA sequences overlapped in CDR3 repertoires of nine tissues is shown in Figure S3. The insertion, splicing regions of the eight CDR3 AA sequences showed multiple identical motif: NT; QNTLY; NERLF (Figure S3).

The number of the top twenty public CDR3 sequences of breast tumor tissues, lung metastatic tissues, and spleens in the three mice was showed in Figures 6(e) and 6(f). Spleens were higher than breast tumor tissues and lung metastatic tissues (Figure 6(e)) but with no difference (Figure 6(f)).

### 3.7. The Usage and Gene Recombination of TRBV and TRBJ

The usage and gene recombination of TRBV and TRBJ in unique productive CDR3 repertoires of three mice are shown in Figure S4 and S5 and S6. The shared TRBV genes used in the three mice were TRBV5-1, TRBV13-1, TRBV13-2, TRBV19-1; and shared TRBJ genes usage were TRBJ1-4, TRBJ2-1, TRBJ2-4, TRBJ2-5, TRBJ2-7. The dominant usage of Mouse 1 and Mouse 3 was TRBV13-02-TRBJ02-07; and Mouse 2 was TRBV19-01-TRBJ02-07.

### 3.8. The Usage and Gene Recombination of TRBV-TRBJ

The gene recombination of TRBV-TRBJ from nine tissue samples was found by dendrogram analysis. Three mice with TRBV-TRBJ gene in spleens were the most similar (Figure S7-1&S7-2&S7-3). The TRBV9-1, TRBV25-1, and TRBV28-1 genes of CDR3 repertoires in Mouse 1 breast tumor tissue and lung metastatic tissue were lost; and the TRBV9-1 gene of CDR3 repertoires in Mouse 2 breast tumor tissue and lung metastatic tissue was lost.

### 3.9. The Characterization and Length of CDR3 AA Repertoires

The length of CDR3 in unique productive CDR3 repertoires of nine tissues was mainly 12 AA dominated Gauss distribution (Figure S8), and the length distribution was concentrated between 8 and 16 AA.

In the CDR3 region, AA was used for the high frequency of amino group hydrophilic amino acids represented by serine (S), and the two ends were more conservative (Figure S9).

## 4. Discussion

Previous studies have confirmed that the number and proportion of CD4^+^CD25^+^Tregs subsets are related to the pathological type, metastasis, and prognosis of breast cancer closely [8–10]. The number of CD4^+^CD25^+^Tregs in patients with breast cancer was found to decrease after chemotherapy or radiotherapy. This decrease was accompanied by enhanced cytotoxicity of NK cells [33, 34]. However, the origin, differentiation, and mechanism of activation of CD4^+^CD25^+^ T cells in breast cancer patients and the homogeneity and heterogeneity of negative regulation of CD4^+^ CD25^+^ T cells remain to be elucidated for breast cancer. In mice, the identification of Tregs as CD4+CD25+ T cells has been used widely. In this study, HTS technology and the immune informatics analysis method were used to analyze the composition and characteristics of the CD4^+^CD25^+^ T cells CDR3 repertoires in breast tumor tissues, lung metastatic tissues, and spleens from 4T1 tumor-bearing BALB/c mice.

A high proportion of CD4^+^CD25^+^ T cells samples in the breast tumor tissues, lung metastatic tissues, and spleens of tumor-bearing mice were separated (Figures 1 and 2). Cell surface markers for CD4^+^CD25^+^ also include a small part activated T cells [3, 4]. However, in mice, the identification of Tregs as CD4^+^CD25^+^ T cells has been used widely, representing approximately 5–10% of CD4+ T cells in the periphery [5]. Moreover, CD4^+^FoxP3^+^CD25^+^ T cells account for 66.7% of CD25^+^CD4^+^ T cells in mouse spleen [6]. Total and unique productive CD4^+^CD25^+^ T cells TCR *β* CDR3 sequence data in nine tissues from three mice were examined using high-throughput sequencing.

Analysis of the CDR3 repertoires indicated that breast tumor tissues, lung metastatic tissues, and spleens from 4T1 tumor-bearing BALB/c mice have visible differences among individuals. Breast tumor tissues showed the least diversity, followed by lung metastatic tissues, and spleens showed the most diversity (Figure 3). These results indicate that there may be more specific clonal CD4^+^CD25^+^ T cells in breast tumor and lung metastatic tissues that are not present in spleens. Moreover, Beausang et al. found that higher proportion of Tregs get activated and expresses immune checkpoints in breast tumor cells. Tregs also expressed the highest levels of other Treg cell-associated molecules, such as Ki-67, HELIOS, CD25, and CD39, which could represent additional druggable targets [35]. Further analysis of the proportion of CDR3 repertoires appearing at high frequency (over 0.05%) and intermediate frequency (0.01–0.05%) and the CDR3 sequence proliferation ratio showed breast tumor tissues and lung metastatic tissues to have greater numbers of both high- and intermediate-frequency sequences than spleens (Figure S2). Low-frequency (under 0.01%) clonal proliferation ratios showed the opposite pattern; spleen tissues had greater numbers of low-frequency sequences than lung metastatic or breast tumor tissues. The results also showed that breast tumor tissues and metastatic tumor tissues had greater frequencies of cloning CD4^+^CD25^+^ T cells, which made it possible to promote tumor growth and metastasis. Olkhanud et al. found that lung metastasis of breast cancer requires high expression of CCR4 Tregs to kill NK cells and inhibition of CCR4^+^ Tregs can reduce the death rate in model mice [36].

Although this experiment did not evaluate the function of CD4^+^CD25^+^ T cells in the high and middle frequencies in tumor tissue or metastatic lung tissues, we did analyze the characteristics and proportion of AA/TRBV/TRBJ of the top five/ten/twenty highest-frequency CDR3s in three mice (Tables 1–10). The overall proportions of the top five/ten/twenty high-frequency CDR3 sequences of breast tumor and lung metastatic tissues were higher than those of spleens. Highly expressed sequences of CDR3 repertoires in three mice with breast tumor tissues and lung metastatic tissues were less in spleens. These results suggested that specific activation of CD4^+^CD25^+^ T cells may take place in breast tumor tissues and lung metastatic tissues. These results are similar to those reported in clinical studies [10]. Both human and mouse studies suggested the diversity and complexity of the source and function of CD4^+^CD25^+^ T cells associated with breast cancer.

The overlap of public CDR3 sequences was more pronounced in spleens than in the other two tissues. Breast tumor tissues shared more publicly known CDR3 sequences with lung metastatic tissues than with spleens (^∗∗^*p* < 0.01) (Figure 4). The results suggested that there may exist some CD4^+^CD25^+^ T cells that promote tumor genesis and metastasis in the tumor primary and metastatic tissues.

On this basis, we analyzed the proportion of eight overlapping CDR3 sequences from nine tissues. Five of them has significant differences between the three tissues (Figures 6(c) and 6(d)). Results suggested that the difference in clonal increments in different tissues may be related to its inhibitory effect. Eight CDR3 sequences of insertion, splicing regions showed multiple identical motifs: NT; QNTLY; NERLF (Figure S3). This suggested that CD4^+^CD25^+^ T cells may share a common motif in response to the same antigen in the 4T1 cell line.

The use of the TRBV and TRBJ gene remained consistent across three mouse tissues. The TRBV9-1, TRBV25-1, and TRBV28-1 genes of the CDR3 repertoires were absent from breast tumor tissue and lung metastatic tissue from Mouse 1; and the TRBV9-1 gene of CDR3 repertoires were absent from breast tumor tissue and lung metastatic tissue from Mouse 2. Wang et al. found that T cells repertoire diversity in breast tumors was higher than that in nontumor tissue, with a preferential use of TRBV and TRBJ genes [37]. The lengths of CDR3 sequences in the unique productive CDR3 repertoires showed 12 AA dominated distribution, and the CDR3 length distribution was concentrated between 8 and 16 AA. In the CDR3 region, AA was used for the high frequency of amino group hydrophilic amino acids represented by serine (S). Results suggested there was no significant difference in the overall characteristics of CD4^+^CD25^+^ T cells CDR3 repertoires in different tissues from 4T1 tumor-bearing BALB/c mice.

In this study, the CD4^+^CD25^+^ T cells CDR3 repertoires in breast tumor tissues and lung metastatic tissues were roughly the same with respect to the diversity of the CDR3 repertoires, high-frequency clone proliferation, composition of the CDR3 and the overlapping ratio, and public CDR3 sequences, but they were different from that in spleens. 4T1 tumor-bearing BALB/c mice showed more intermediate- and high-frequency CD4^+^CD25^+^ T cells CDR3 repertoires; there were also some specific CD4^+^CD25^+^ T cells that showed high-frequency proliferation. Because FoxP3 was not detected, CD4^+^CD25^+^ T cells may contain partially activated T cells. However, these results were consistent with the changes in CD4^+^CD25^+^ Tregs in the clinical treatment of breast cancer in recent years. The number of Tregs was significantly reduced by letrozole treated in the elderly patients with ER(+) breast cancer [38]. The quantity of peripheral blood Tregs in breast cancer patients was found to decrease after treatment with herceptin [39]. Treatment with Trastuzumab was associated with a decrease in the ratio of CD4^+^CD25^+^ Tregs in peripheral blood and a decrease in the quantity of Tregs [8]. The number of Th17 cells increased [40]. These therapies may reduce the median frequency of Tregs by killing and inhibiting tumor growth. Clinical research reported by Schmidt et al. showed that breast globin and HLA-DRB1 ^∗^04 : 01; HLA-DRB1 ^∗^07 : 01 as prepared by Tetramer Technology can detect the specific Tregs in breast cancer patients [41]. Gates et al. used HLA II molecules with HER2/neu peptide (AE37) vaccine treatment on breast cancer patients and found that the number of peripheral blood lymphocytes CD4^+^CD25^+^Foxp3^+^Tregs decreased in the patients [42]. This suggested that breast cancer patients may have high frequencies of Tregs, which can be used as a therapeutic target for breast cancer patients with high-frequency specific Tregs. Recently, Page *et al.* found the changes in TIL CDR3 repertoires to be associated with immunotherapy (cryoablation, single-dose anti-CTLA-4 (ipilimumab, or cryoablation, and ipilimumab) in breast cancer patients [43]. The results suggested that the TIL CDR3 repertoires can be used as an evaluation index for the treatment of early breast cancer patients. In addition, Núñez et al. studied tumor-draining lymph node (TDLN) invasion by metastatic cells in breast cancer and found that TDLN Tregs were functional and express a distinct pattern of druggable coreceptors, highlighting their potential as targets for cancer immunotherapy [44]. Combined with the results of these previous clinical studies, the data reported here may be used to further explore the characteristics of the CDR3 repertoires and assess the breast cancer microenvironment of CD4^+^CD25^+^Tregs.

## Figures and Tables

**Table 1:** Analysis of composition and characteristics of frequency for the top five sequences and the usage of TRBV and TRBJ gene, CDR3 AA of CD4^+^CD25^+^ T cells TCR *β* CDR3 repertoires in breast tumor tissues, lung metastatic tissues, and spleens from 4T1 tumor-bearing BALB/c mouse 1.

	TRV gene	TRJ gene	CDR3 sequences (AA)	Frequency (%)	Total frequency (%)
M1-B (top5)	TCRBV31-01	TCRBJ01-05	CAWRGTFHNNQAPLF	1.181	3.983
	TCRBV13-01	TCRBJ02-07	CASSERLGGYEQYF	1.099	
	TCRBV02-01	TCRBJ01-05	CASSQGQGANQAPLF	0.741	
	TCRBV29-01	TCRBJ01-02	CASSLGTGNSDYTF	0.483	
	TCRBV31-01	TCRBJ02-07	CAWSPGLGGRDEQYF	0.479	

M1-L (top5)	TCRBV13-01	TCRBJ02-07	CASSERLGGYEQYF	0.531	1.980
	TCRBV02-01	TCRBJ01-05	CASSQGQGANQAPLF	0.427	
	TCRBV31-01	TCRBJ01-05	CAWRGTFHNNQAPLF	0.375	
	TCRBV13-02	TCRBJ02-01	CASGGDWGGNYAEQFF	0.323	
	TCRBV13-02	TCRBJ01-04	CASGDISNERLFF	0.323	

M1-S (top5)	TCRBV20-01	TCRBJ02-01	CGARPWGGAEQFF	0.309	0.769
	TCRBV31-01	TCRBJ01-05	CAWRGTFHNNQAPLF	0.159	
	TCRBV19-01	TCRBJ01-04	CASSTRTSNERLFF	0.109	
	TCRBV04-01	TCRBJ02-01	CASSFRDRGYAEQFF	0.100	
	TCRBV17-01	TCRBJ01-03	CASSSRTGSGNTLYF	0.092	

**Table 2:** Analysis of composition and characteristics of frequency for the top five sequences and the usage of TRBV and TRBJ gene, CDR3 AA of CD4^+^CD25^+^ T cells TCR *β* CDR3 repertoires in breast tumor tissues, lung metastatic tissues, and spleens from 4T1 tumor-bearing BALB/c mouse 2.

Sample	TRV gene	TRJ gene	CDR3 sequences (AA)	Frequency (%)	Total frequency (%)
M2-B (top5)	TCRBV02-01	TCRBJ02-07	CASSPGHYEQYF	0.467	1.497
	TCRBV02-01	TCRBJ02-07	CASSQAGTGVYEQYF	0.339	
	TCRBV14-01	TCRBJ02-07	CASRGGGYEQYF	0.252	
	TCRBV13-01	TCRBJ01-02	CASRTANSDYTF	0.223	
	TCRBV24-01	TCRBJ02-07	CASSLGLGDYEQYF	0.215	

M2-L (top5)	TCRBV16-01	TCRBJ02-05	CASSSWGGQDTQYF	0.825	2.768
	TCRBV02-01	TCRBJ02-07	CASSQAGTGVYEQYF	0.770	
	TCRBV14-01	TCRBJ01-02	CASSQRTDANSDYTF	0.440	
	TCRBV31-01	TCRBJ02-03	CAWSPGTGGGAETLYF	0.422	
	TCRBV13-02	TCRBJ01-04	CASGDEGHSNERLFF	0.312	

M2-S (top5)	TCRBV02-01	TCRBJ02-07	CASSPGHYEQYF	0.202	0.696
	TCRBV02-01	TCRBJ02-02	CASSQGDRVTGQLYF	0.143	
	TCRBV13-03	TCRBJ02-07	CASRGPGQGGEQYF	0.119	
	TCRBV02-01	TCRBJ02-07	CASSQGLGGSEQYF	0.119	
	TCRBV19-01	TCRBJ01-05	CASTPGRNNQAPLF	0.114	

**Table 3:** Analysis of composition and characteristics of frequency for the top five sequences and the usage of TRBV and TRBJ gene, CDR3 AA of CD4^+^CD25^+^ T cells TCR *β* CDR3 repertoires in breast tumor tissues, lung metastatic tissues, and spleens from 4T1 tumor-bearing BALB/c mouse 3.

Sample	TRV gene	TRJ gene	CDR3 sequences (AA)	Frequency (%)	Total frequency (%)
M3-B (top5)	TCRBV19-01	TCRBJ01-03	CASSTSSGNTLYF	1.217	2.936
	TCRBV31-01	TCRBJ02-03	CAWSPGLGGSAETLYF	0.908	
	TCRBV13-02	TCRBJ02-07	CASGSAGGISYEQYF	0.344	
	TCRBV13-01	TCRBJ02-07	CASSEPGGIYEQYF	0.282	
	TCRBV19-01	TCRBJ02-01	CASSIKLGGYAEQFF	0.186	

M3-L (top5)	TCRBV19-01	TCRBJ01-03	CASSTSSGNTLYF	1.629	3.160
	TCRBV31-01	TCRBJ02-03	CAWSPGLGGSAETLYF	0.504	
	TCRBV13-03	TCRBJ02-07	CASSEGLGGVKQYF	0.407	
	TCRBV13-01	TCRBJ02-07	CASSEPGGIYEQYF	0.388	
	TCRBV13-01	TCRBJ02-07	CASSDATGGATYEQYF	0.233	

M3-S (top5)	TCRBV13-01	TCRBJ02-07	CASSEPGGIYEQYF	0.137	0.592
	TCRBV19-01	TCRBJ01-03	CASSTSSGNTLYF	0.128	
	TCRBV13-02	TCRBJ02-07	CASGSAGGISYEQYF	0.124	
	TCRBV19-01	TCRBJ02-01	CASSKGQGRYAEQFF	0.106	
	TCRBV13-03	TCRBJ02-07	CASSEGLGGVKQYF	0.097	

**Table 4:** The number of overlapped CDR3 sequences of CD4^+^CD25^+^ T cells TCR *β* CDR3 repertoires in breast tumor tissues, lung metastatic tissues, and spleens from 4T1 tumor-bearing BALB/c mice.

	Tissue type	Top 5	Top 10	Top 20
M1	B/L	3	6	8
	B/S	1	1	5
	L/S	1	1	2

M2	B/L	1	1	2
	B/S	1	1	4
	L/S	1	1	2

M3	B/L	3	4	5
	B/S	3	5	8
	L/S	2	3	3

**Table 5:** Analysis of composition and characteristics of frequency for the top ten sequences and the usage of TRBV and TRBJ gene, CDR3 AA of CD4^+^CD25^+^ T cells TCR *β* CDR3 repertoires in breast tumor tissues, lung metastatic tissues, and spleens from 4T1 tumor-bearing BALB/c mouse 1.

Sample	TRV gene	TRJ gene	CDR3 sequences (AA)	Frequency (%)	Total frequency (%)
M1-B (top10)	TCRBV31-01	TCRBJ01-05	CAWRGTFHNNQAPLF	1.181	4.979
	TCRBV13-01	TCRBJ02-07	CASSERLGGYEQYF	1.099	
	TCRBV02-01	TCRBJ01-05	CASSQGQGANQAPLF	0.741	
	TCRBV29-01	TCRBJ01-02	CASSLGTGNSDYTF	0.483	
	TCRBV31-01	TCRBJ02-07	CAWSPGLGGRDEQYF	0.479	
	TCRBV20-01	TCRBJ02-07	CGARTGQGSYEQYF	0.239	
	TCRBV31-01	TCRBJ02-02	CAWKATGNTGQLYF	0.224	
	TCRBV19-01	TCRBJ02-05	CASRDREGNQDTQYF	0.210	
	TCRBV26-01	TCRBJ01-06	LCQQSTGSYNSPLYF	0.198	
	TCRBV19-01	TCRBJ01-03	CASSTYRGLSGNTLYF	0.125	

M1-L (top10)	TCRBV13-01	TCRBJ02-07	CASSERLGGYEQYF	0.531	2.917
	TCRBV02-01	TCRBJ01-05	CASSQGQGANQAPLF	0.427	
	TCRBV31-01	TCRBJ01-05	CAWRGTFHNNQAPLF	0.375	
	TCRBV13-02	TCRBJ02-01	CASGGDWGGNYAEQFF	0.323	
	TCRBV13-02	TCRBJ01-04	CASGDISNERLFF	0.323	
	TCRBV13-02	TCRBJ01-05	CASGDTNNQAPLF	0.198	
	TCRBV20-01	TCRBJ02-07	CGARTGQGSYEQYF	0.198	
	TCRBV31-01	TCRBJ02-07	CAWSPGLGGRDEQYF	0.198	
	TCRBV19-01	TCRBJ01-04	CASSLDRINERLFF	0.177	
	TCRBV29-01	TCRBJ01-02	CASSLGTGNSDYTF	0.167	

M1-S (top10)	TCRBV20-01	TCRBJ02-01	CGARPWGGAEQFF	0.309	1.161
	TCRBV31-01	TCRBJ01-05	CAWRGTFHNNQAPLF	0.159	
	TCRBV19-01	TCRBJ01-04	CASSTRTSNERLFF	0.109	
	TCRBV04-01	TCRBJ02-01	CASSFRDRGYAEQFF	0.100	
	TCRBV17-01	TCRBJ01-03	CASSSRTGSGNTLYF	0.092	
	TCRBV31-01	TCRBJ02-07	CAWSLRTGGSSYEQYF	0.084	
	TCRBV13-03	TCRBJ01-05	CASRGTGNNQAPLF	0.084	
	TCRBV13-03	TCRBJ02-07	CASSDAGWGEGQYF	0.075	
	TCRBV13-02	TCRBJ02-01	CASGGDWGGNYAEQFF	0.075	
	TCRBV17-01	TCRBJ02-07	CASSRGPGTGYEQYF	0.075	

**Table 6:** Analysis of composition and characteristics of frequency for the top ten sequences and the usage of TBBV and TRBJ gene, CDR3 AA of CD4^+^CD25^+^ T cells TCR *β* CDR3 repertoires in breast tumor tissues, lung metastatic tissues, and spleens from 4T1 tumor-bearing BALB/c mouse 2.

Sample	TRV gene	TRJ gene	CDR3 sequences (AA)	Frequency (%)	Total frequency (%)
M2-B (top10)	TCRBV02-01	TCRBJ02-07	CASSPGHYEQYF	0.467	2.489
	TCRBV02-01	TCRBJ02-07	CASSQAGTGVYEQYF	0.339	
	TCRBV14-01	TCRBJ02-07	CASRGGGYEQYF	0.252	
	TCRBV13-01	TCRBJ01-02	CASRTANSDYTF	0.223	
	TCRBV24-01	TCRBJ02-07	CASSLGLGDYEQYF	0.215	
	TCRBV19-01	TCRBJ02-07	CASSSGTGAYEQYF	0.215	
	TCRBV05-01	TCRBJ02-07	CASSQVDWGGSYEQYF	0.198	
	TCRBV19-01	TCRBJ02-07	CASRPGLGGYEQYF	0.198	
	TCRBV05-01	TCRBJ02-07	CASSQEGVSYEQYF	0.194	
	TCRBV16-01	TCRBJ02-07	CASSLETGAYEQYF	0.186	

M2-L (top10)	TCRBV16-01	TCRBJ02-05	CASSSWGGQDTQYF	0.825	4.069
	TCRBV02-01	TCRBJ02-07	CASSQAGTGVYEQYF	0.770	
	TCRBV14-01	TCRBJ01-02	CASSQRTDANSDYTF	0.440	
	TCRBV31-01	TCRBJ02-03	CAWSPGTGGGAETLYF	0.422	
	TCRBV13-02	TCRBJ01-04	CASGDEGHSNERLFF	0.312	
	TCRBV20-01	TCRBJ01-04	CGAGDRGPNERLFF	0.293	
	TCRBV01-01	TCRBJ02-07	CTCSAGQSSYEQYF	0.293	
	TCRBV19-01	TCRBJ02-07	CASSTGSSYEQYF	0.275	
	TCRBV05-01	TCRBJ02-03	CASSQENWGSAETLYF	0.220	
	TCRBV19-01	TCRBJ02-01	CASSSRTGGYAEQFF	0.220	

M2-S (top10)	TCRBV02-01	TCRBJ02-07	CASSPGHYEQYF	0.202	1.205
	TCRBV02-01	TCRBJ02-02	CASSQGDRVTGQLYF	0.143	
	TCRBV13-03	TCRBJ02-07	CASRGPGQGGEQYF	0.119	
	TCRBV02-01	TCRBJ02-07	CASSQGLGGSEQYF	0.119	
	TCRBV19-01	TCRBJ01-05	CASTPGRNNQAPLF	0.114	
	TCRBV31-01	TCRBJ01-03	CAQGQHLNSGNTLYF	0.114	
	TCRBV02-01	TCRBJ02-07	CASSQAGTGVYEQYF	0.104	
	TCRBV05-01	TCRBJ02-07	CASSQDGGRTYEQYF	0.104	
	TCRBV02-01	TCRBJ02-07	CASSQNPGQGAYEQYF	0.099	
	TCRBV05-01	TCRBJ01-05	CASSQGNNQAPLF	0.089	

**Table 7:** Analysis of composition and characteristics of frequency for the top ten sequences and the usage of TBBV and TRBJ gene, CDR3 AA of CD4^+^CD25^+^ T cells TCR *β* CDR3 repertoires in breast tumor tissues, lung metastatic tissues, and spleens from 4T1 tumor-bearing BALB/c mouse 3.

Sample	TRV gene	TRJ gene	CDR3 sequences (AA)	Frequency (%)	Total frequency (%)
M3-B (top10)	TCRBV19-01	TCRBJ01-03	CASSTSSGNTLYF	1.217	3.575
	TCRBV31-01	TCRBJ02-03	CAWSPGLGGSAETLYF	0.908	
	TCRBV13-02	TCRBJ02-07	CASGSAGGISYEQYF	0.344	
	TCRBV13-01	TCRBJ02-07	CASSEPGGIYEQYF	0.282	
	TCRBV19-01	TCRBJ02-01	CASSIKLGGYAEQFF	0.186	
	TCRBV13-03	TCRBJ02-07	CASSEGLGGVKQYF	0.158	
	TCRBV19-01	TCRBJ02-01	CASSKGQGRYAEQFF	0.138	
	TCRBV02-01	TCRBJ01-03	CASRPGQTGNTLYF	0.117	
	TCRBV19-01	TCRBJ01-05	CASSLSGLWRAPLF	0.117	
	TCRBV13-01	TCRBJ02-07	CASSETGTEQYF	0.110	

M3-L (top10)	TCRBV19-01	TCRBJ01-03	CASSTSSGNTLYF	1.629	4.091
	TCRBV31-01	TCRBJ02-03	CAWSPGLGGSAETLYF	0.504	
	TCRBV13-03	TCRBJ02-07	CASSEGLGGVKQYF	0.407	
	TCRBV13-01	TCRBJ02-07	CASSEPGGIYEQYF	0.388	
	TCRBV13-01	TCRBJ02-07	CASSDATGGATYEQYF	0.233	
	TCRBV13-02	TCRBJ02-07	CASGSAGGISYEQYF	0.233	
	TCRBV04-01	TCRBJ02-07	CASSGGVEQYF	0.194	
	TCRBV19-01	TCRBJ02-01	CASSIKLGGYAEQFF	0.174	
	TCRBV13-02	TCRBJ01-03	CASGETTNSGNTLYF	0.174	
	TCRBV20-01	TCRBJ01-02	CGARDNANSDYTF	0.155	

M3-S (top10)	TCRBV13-01	TCRBJ02-07	CASSEPGGIYEQYF	0.137	0.998
	TCRBV19-01	TCRBJ01-03	CASSTSSGNTLYF	0.128	
	TCRBV13-02	TCRBJ02-07	CASGSAGGISYEQYF	0.124	
	TCRBV19-01	TCRBJ02-01	CASSKGQGRYAEQFF	0.106	
	TCRBV13-03	TCRBJ02-07	CASSEGLGGVKQYF	0.097	
	TCRBV13-02	TCRBJ01-04	CASGDWNERLFF	0.088	
	TCRBV31-01	TCRBJ01-01	CAWSPPTANTEVFF	0.084	
	TCRBV14-01	TCRBJ02-07	CASSLTGGEVEQYF	0.084	
	TCRBV31-01	TCRBJ01-03	CAWSRQVNSGNTLYF	0.084	
	TCRBV02-01	TCRBJ01-04	CASSAGRPNERLFF	0.066	

**Table 8:** Analysis of composition and characteristics of frequency for the top twenty sequences and the usage of TRBV and TRBJ gene, CDR3 AA of CD4^+^CD25^+^ T cells TCR *β* CDR3 repertoires in breast tumor tissues, lung metastatic tissues, and spleens from 4T1 tumor-bearing BALB/c mouse 1.

Sample	TRV gene	TRJ gene	CDR3 sequences (AA)	Frequency (%)	Total frequency (%)
M1-B (top20)	TCRBV31-01	TCRBJ01-05	CAWRGTFHNNQAPLF	1.181	6.043
	TCRBV13-01	TCRBJ02-07	CASSERLGGYEQYF	1.099	
	TCRBV02-01	TCRBJ01-05	CASSQGQGANQAPLF	0.741	
	TCRBV29-01	TCRBJ01-02	CASSLGTGNSDYTF	0.483	
	TCRBV31-01	TCRBJ02-07	CAWSPGLGGRDEQYF	0.479	
	TCRBV20-01	TCRBJ02-07	CGARTGQGSYEQYF	0.239	
	TCRBV31-01	TCRBJ02-02	CAWKATGNTGQLYF	0.224	
	TCRBV19-01	TCRBJ02-05	CASRDREGNQDTQYF	0.210	
	TCRBV26-01	TCRBJ01-06	LCQQSTGSYNSPLYF	0.198	
	TCRBV19-01	TCRBJ01-03	CASSTYRGLSGNTLYF	0.125	
	TCRBV31-01	TCRBJ02-07	CAWSLRTGGSSYEQYF	0.123	
	TCRBV05-01	TCRBJ01-04	CASSQEGGEGERLFF	0.116	
	TCRBV20-01	TCRBJ02-01	CGARPWGGAEQFF	0.111	
	TCRBV02-01	TCRBJ01-05	CASSQETDRGQAPLF	0.111	
	TCRBV19-01	TCRBJ02-04	CASSIWDDQNTLYF	0.111	
	TCRBV19-01	TCRBJ01-04	CASSLDRINERLFF	0.109	
	TCRBV17-01	TCRBJ01-04	CASSRRGQGISNERLFF	0.104	
	TCRBV02-01	TCRBJ01-03	CASSPMTGTGNTLYF	0.094	
	TCRBV13-02	TCRBJ01-03	CASGDARNSGNTLYF	0.094	
	TCRBV19-01	TCRBJ01-04	CASSTRTSNERLFF	0.092	

M1-L (top20)	TCRBV13-01	TCRBJ02-07	CASSERLGGYEQYF	0.531	4.209
	TCRBV02-01	TCRBJ01-05	CASSQGQGANQAPLF	0.427	
	TCRBV31-01	TCRBJ01-05	CAWRGTFHNNQAPLF	0.375	
	TCRBV13-02	TCRBJ02-01	CASGGDWGGNYAEQFF	0.323	
	TCRBV13-02	TCRBJ01-04	CASGDISNERLFF	0.323	
	TCRBV13-02	TCRBJ01-05	CASGDTNNQAPLF	0.198	
	TCRBV20-01	TCRBJ02-07	CGARTGQGSYEQYF	0.198	
	TCRBV31-01	TCRBJ02-07	CAWSPGLGGRDEQYF	0.198	
	TCRBV19-01	TCRBJ01-04	CASSLDRINERLFF	0.177	
	TCRBV29-01	TCRBJ01-02	CASSLGTGNSDYTF	0.167	
	TCRBV31-01	TCRBJ02-07	CAWSLRTGGSSYEQYF	0.156	
	TCRBV14-01	TCRBJ01-05	CASSLRDRGQAPLF	0.135	
	TCRBV01-01	TCRBJ01-03	CTCSGTGGSGNTLYF	0.135	
	TCRBV13-02	TCRBJ02-07	CASGDVGLSSYEQYF	0.135	
	TCRBV31-01	TCRBJ02-01	CAWSLFGGNYAEQFF	0.125	
	TCRBV20-01	TCRBJ02-01	CGARPWGGAEQFF	0.125	
	TCRBV05-01	TCRBJ02-01	CASSQEGGWGNYAEQFF	0.125	
	TCRBV13-02	TCRBJ02-07	CASGDAGGAYEQYF	0.125	
	TCRBV17-01	TCRBJ02-07	CASSRTGGSYEQYF	0.115	
	TCRBV02-01	TCRBJ01-05	CASSQETDRGQAPLF	0.115	

M1-S (top20)	TCRBV20-01	TCRBJ02-01	CGARPWGGAEQFF	0.309	1.847
	TCRBV31-01	TCRBJ01-05	CAWRGTFHNNQAPLF	0.159	
	TCRBV19-01	TCRBJ01-04	CASSTRTSNERLFF	0.109	
	TCRBV04-01	TCRBJ02-01	CASSFRDRGYAEQFF	0.100	
	TCRBV17-01	TCRBJ01-03	CASSSRTGSGNTLYF	0.092	
	TCRBV31-01	TCRBJ02-07	CAWSLRTGGSSYEQYF	0.084	
	TCRBV13-03	TCRBJ01-05	CASRGTGNNQAPLF	0.084	
	TCRBV13-03	TCRBJ02-07	CASSDAGWGEGQYF	0.075	
	TCRBV13-02	TCRBJ02-01	CASGGDWGGNYAEQFF	0.075	
	TCRBV17-01	TCRBJ02-07	CASSRGPGTGYEQYF	0.075	
	TCRBV14-01	TCRBJ02-07	CASSFGDKYEQYF	0.075	
	TCRBV29-01	TCRBJ02-01	CASSLSMVGQFF	0.075	
	TCRBV31-01	TCRBJ02-01	CAWSLFGGNYAEQFF	0.067	
	TCRBV02-01	TCRBJ01-03	CASSTGVGNTLYF	0.067	
	TCRBV19-01	TCRBJ01-03	CASSSGVGNTLYF	0.067	
	TCRBV02-01	TCRBJ02-07	CASSQDSSYEQYF	0.067	
	TCRBV19-01	TCRBJ01-04	CASSIRTGNERLFF	0.067	
	TCRBV14-01	TCRBJ02-01	CASRRSYAEQFF	0.067	
	TCRBV13-02	TCRBJ01-03	CASGDARNSGNTLYF	0.067	
	TCRBV13-02	TCRBJ01-05	CASGDQQAPLF	0.067	

**Table 9:** Analysis of composition and characteristics of frequency for the top twenty sequences and the usage of TRBV and TRBJ gene, CDR3 AA of CD4^+^CD25^+^ T cells TCR *β* CDR3 repertoires in breast tumor tissues, lung metastatic tissues, and spleens from 4T1 tumor-bearing BALB/c mouse 2.

Sample	TRV gene	TRJ gene	CDR3 sequences (AA)	Frequency (%)	Total frequency (%)
M2-B (top20)	TCRBV02-01	TCRBJ02-07	CASSPGHYEQYF	0.467	3.899
	TCRBV02-01	TCRBJ02-07	CASSQAGTGVYEQYF	0.339	
	TCRBV14-01	TCRBJ02-07	CASRGGGYEQYF	0.252	
	TCRBV13-01	TCRBJ01-02	CASRTANSDYTF	0.223	
	TCRBV24-01	TCRBJ02-07	CASSLGLGDYEQYF	0.215	
	TCRBV19-01	TCRBJ02-07	CASSSGTGAYEQYF	0.215	
	TCRBV05-01	TCRBJ02-07	CASSQVDWGGSYEQYF	0.198	
	TCRBV19-01	TCRBJ02-07	CASRPGLGGYEQYF	0.198	
	TCRBV05-01	TCRBJ02-07	CASSQEGVSYEQYF	0.194	
	TCRBV16-01	TCRBJ02-07	CASSLETGAYEQYF	0.186	
	TCRBV19-01	TCRBJ02-07	CASSMGGLGYEQYF	0.174	
	TCRBV05-01	TCRBJ02-07	CASSQGHYEQYF	0.157	
	TCRBV02-01	TCRBJ02-07	CASSQNPGQGAYEQYF	0.149	
	TCRBV20-01	TCRBJ01-05	CGAPGQGNQAPLF	0.145	
	TCRBV01-01	TCRBJ01-05	CTCSEQGTNQAPLF	0.145	
	TCRBV19-01	TCRBJ01-05	CASTPGRNNQAPLF	0.145	
	TCRBV02-01	TCRBJ02-07	CASSQPAGSYEQYF	0.128	
	TCRBV19-01	TCRBJ02-07	CASSIEGRGVYEQYF	0.124	
	TCRBV26-01	TCRBJ02-07	LCQQSSPVYEQYF	0.124	
	TCRBV05-01	TCRBJ02-01	CASSQDWGDTYAEQFF	0.120	

M2-L (top20)	TCRBV16-01	TCRBJ02-05	CASSSWGGQDTQYF	0.825	6.012
	TCRBV02-01	TCRBJ02-07	CASSQAGTGVYEQYF	0.770	
	TCRBV14-01	TCRBJ01-02	CASSQRTDANSDYTF	0.440	
	TCRBV31-01	TCRBJ02-03	CAWSPGTGGGAETLYF	0.422	
	TCRBV13-02	TCRBJ01-04	CASGDEGHSNERLFF	0.312	
	TCRBV20-01	TCRBJ01-04	CGAGDRGPNERLFF	0.293	
	TCRBV01-01	TCRBJ02-07	CTCSAGQSSYEQYF	0.293	
	TCRBV19-01	TCRBJ02-07	CASSTGSSYEQYF	0.275	
	TCRBV05-01	TCRBJ02-03	CASSQENWGSAETLYF	0.220	
	TCRBV19-01	TCRBJ02-01	CASSSRTGGYAEQFF	0.220	
	TCRBV01-01	TCRBJ02-01	CTCSADPAHYAEQFF	0.220	
	TCRBV24-01	TCRBJ02-07	CASSLWGYEQYF	0.220	
	TCRBV12-01	TCRBJ01-02	CASSLPGTGDSDYTF	0.220	
	TCRBV13-03	TCRBJ01-03	CASSDAGNSGNTLYF	0.202	
	TCRBV05-01	TCRBJ02-07	CASSQDWGGASYEQYF	0.183	
	TCRBV04-01	TCRBJ02-07	CASSYSEYEQYF	0.183	
	TCRBV26-01	TCRBJ02-07	LCQQSSPVYEQYF	0.183	
	TCRBV05-01	TCRBJ02-07	CASSQDGGRTYEQYF	0.183	
	TCRBV19-01	TCRBJ02-03	CASSVGGNAETLYF	0.183	
	TCRBV24-01	TCRBJ01-03	CASSCSSGNTLYF	0.165	

M2-S (top20)	TCRBV02-01	TCRBJ02-07	CASSPGHYEQYF	0.202	2.005
	TCRBV02-01	TCRBJ02-02	CASSQGDRVTGQLYF	0.143	
	TCRBV13-03	TCRBJ02-07	CASRGPGQGGEQYF	0.119	
	TCRBV02-01	TCRBJ02-07	CASSQGLGGSEQYF	0.119	
	TCRBV19-01	TCRBJ01-05	CASTPGRNNQAPLF	0.114	
	TCRBV31-01	TCRBJ01-03	CAQGQHLNSGNTLYF	0.114	
	TCRBV02-01	TCRBJ02-07	CASSQAGTGVYEQYF	0.104	
	TCRBV05-01	TCRBJ02-07	CASSQDGGRTYEQYF	0.104	
	TCRBV02-01	TCRBJ02-07	CASSQNPGQGAYEQYF	0.099	
	TCRBV05-01	TCRBJ01-05	CASSQGNNQAPLF	0.089	
	TCRBV13-01	TCRBJ01-03	CASSDAGQNSGNTLYF	0.089	
	TCRBV05-01	TCRBJ02-07	CASSQEMGAYEQYF	0.084	
	TCRBV26-01	TCRBJ02-07	LCQQSSPVYEQYF	0.084	
	TCRBV17-01	TCRBJ02-07	CASSRLGASYEQYF	0.084	
	TCRBV02-01	TCRBJ01-03	CASSQDRAGNTLYF	0.079	
	TCRBV01-01	TCRBJ02-01	CTCSADPAHYAEQFF	0.079	
	TCRBV19-01	TCRBJ02-07	CASSTGSSYEQYF	0.079	
	TCRBV02-01	TCRBJ02-02	CASSQGDTGQLYF	0.074	
	TCRBV19-01	TCRBJ01-05	CASRGANNQAPLF	0.074	
	TCRBV04-01	TCRBJ01-03	CASSLGQSGNTLYF	0.074	

**Table 10:** Analysis of composition and characteristics of frequency for the top twenty sequences and the usage of TRBV and TRBJ gene, CDR3 AA of CD4^+^CD25^+^ T cells TCR *β* CDR3 repertoires in breast tumor tissues, lung metastatic tissues, and spleens from 4T1 tumor-bearing BALB/c mouse 3.

Sample	TRV gene	TRJ gene	CDR3 sequences (AA)	Frequency (%)	Total frequency (%)
M3-B (top20)	TCRBV19-01	TCRBJ01-03	CASSTSSGNTLYF	1.217	4.441
	TCRBV31-01	TCRBJ02-03	CAWSPGLGGSAETLYF	0.908	
	TCRBV13-02	TCRBJ02-07	CASGSAGGISYEQYF	0.344	
	TCRBV13-01	TCRBJ02-07	CASSEPGGIYEQYF	0.282	
	TCRBV19-01	TCRBJ02-01	CASSIKLGGYAEQFF	0.186	
	TCRBV13-03	TCRBJ02-07	CASSEGLGGVKQYF	0.158	
	TCRBV19-01	TCRBJ02-01	CASSKGQGRYAEQFF	0.138	
	TCRBV02-01	TCRBJ01-03	CASRPGQTGNTLYF	0.117	
	TCRBV19-01	TCRBJ01-05	CASSLSGLWRAPLF	0.117	
	TCRBV13-01	TCRBJ02-07	CASSETGTEQYF	0.110	
	TCRBV13-02	TCRBJ02-01	CASGGGRNYAEQFF	0.103	
	TCRBV31-01	TCRBJ01-03	CAWSRQVNSGNTLYF	0.089	
	TCRBV01-01	TCRBJ02-07	CTCSAVGGAREQYF	0.089	
	TCRBV20-01	TCRBJ02-07	CGATRDSSYEQYF	0.089	
	TCRBV19-01	TCRBJ01-04	CASSIVGISNERLFF	0.089	
	TCRBV14-01	TCRBJ01-03	CASSFTGAGNTLYF	0.083	
	TCRBV29-01	TCRBJ02-07	CALGQGYEQYF	0.083	
	TCRBV04-01	TCRBJ02-07	CASSGGVEQYF	0.083	
	TCRBV29-01	TCRBJ02-07	CASSLSGQGGEQYF	0.083	
	TCRBV01-01	TCRBJ01-04	CTCSASVSNERLFF	0.076	

M3-L (top20)	TCRBV19-01	TCRBJ01-03	CASSTSSGNTLYF	1.629	5.370
	TCRBV31-01	TCRBJ02-03	CAWSPGLGGSAETLYF	0.504	
	TCRBV13-03	TCRBJ02-07	CASSEGLGGVKQYF	0.407	
	TCRBV13-01	TCRBJ02-07	CASSEPGGIYEQYF	0.388	
	TCRBV13-01	TCRBJ02-07	CASSDATGGATYEQYF	0.233	
	TCRBV13-02	TCRBJ02-07	CASGSAGGISYEQYF	0.233	
	TCRBV04-01	TCRBJ02-07	CASSGGVEQYF	0.194	
	TCRBV19-01	TCRBJ02-01	CASSIKLGGYAEQFF	0.174	
	TCRBV13-02	TCRBJ01-03	CASGETTNSGNTLYF	0.174	
	TCRBV20-01	TCRBJ01-02	CGARDNANSDYTF	0.155	
	TCRBV13-02	TCRBJ02-01	CASGGGRNYAEQFF	0.136	
	TCRBV20-01	TCRBJ01-02	CGARDNANSDYTF	0.136	
	TCRBV02-01	TCRBJ02-07	CASSQDRSSYEQYF	0.136	
	TCRBV02-01	TCRBJ02-07	CASSHRDWGYEQYF	0.136	
	TCRBV05-01	TCRBJ01-05	CASSQDDQAPLF	0.136	
	TCRBV13-01	TCRBJ01-05	CASSGSNNQAPLF	0.136	
	TCRBV20-01	TCRBJ01-03	CGARDNSGNTLYF	0.116	
	TCRBV13-01	TCRBJ01-05	CASSGAGTENQAPLF	0.116	
	TCRBV02-01	TCRBJ01-03	CASSQDHIGNTLYF	0.116	
	TCRBV02-01	TCRBJ02-07	CASSQGDSSYEQYF	0.116	

M3-S (top20)	TCRBV13-01	TCRBJ02-07	CASSEPGGIYEQYF	0.137	1.572
	TCRBV19-01	TCRBJ01-03	CASSTSSGNTLYF	0.128	
	TCRBV13-02	TCRBJ02-07	CASGSAGGISYEQYF	0.124	
	TCRBV19-01	TCRBJ02-01	CASSKGQGRYAEQFF	0.106	
	TCRBV13-03	TCRBJ02-07	CASSEGLGGVKQYF	0.097	
	TCRBV13-02	TCRBJ01-04	CASGDWNERLFF	0.088	
	TCRBV31-01	TCRBJ01-01	CAWSPPTANTEVFF	0.084	
	TCRBV14-01	TCRBJ02-07	CASSLTGGEVEQYF	0.084	
	TCRBV31-01	TCRBJ01-03	CAWSRQVNSGNTLYF	0.084	
	TCRBV02-01	TCRBJ01-04	CASSAGRPNERLFF	0.066	
	TCRBV13-02	TCRBJ01-03	CASGETTNSGNTLYF	0.066	
	TCRBV01-01	TCRBJ02-07	CTCSARGLSYEQYF	0.066	
	TCRBV19-01	TCRBJ01-05	CASSLSGLWRAPLF	0.062	
	TCRBV20-01	TCRBJ01-03	CGARDNSGNTLYF	0.057	
	TCRBV04-01	TCRBJ02-07	CASSYWGGSYEQYF	0.057	
	TCRBV13-02	TCRBJ02-07	CASGGTGVYEQYF	0.057	
	TCRBV01-01	TCRBJ02-07	CTCSAVGGAREQYF	0.057	
	TCRBV19-01	TCRBJ01-04	CASSIVGISNERLFF	0.053	
	TCRBV13-02	TCRBJ02-02	CASGEWGKNTGQLYF	0.049	
	TCRBV02-01	TCRBJ02-01	CASSQEEGNYAEQFF	0.049	

## Data Availability

The data can be accessed at https://data.mendeley.com/datasets/jn7pt7y74d/draft?a=437968bf-1bc5-4061-bd75-3a5c65b8157e.

## Supplementary Materials

Supplementary Materials Figure S1: total DNA 1% agarose gel electrophoresis and DNA concentration. Figure S2: the mean clone percentage and statistical analysis of high-frequency (>0.05%), intermediate-frequency (0.01–0.05%), and low frequency (<0.01%) CD4^+^CD25^+^ T cells TCR *β* CDR3 AA repertoire in three same tissues from three 4T1 tumor-bearing BALB/c mice. Figure S3: the ratio of cloning proliferation of total overlap eight CDR3 sequences from CDR3 repertoire in each tissues among breast tumor tissues, lung metastatic tissues, and spleens from three 4T1 tumor-bearing BALB/c mice (total of nine tissues). Figure S4: the distribution of the usage of TRBV gene of CD4^+^CD25^+^ T cells in breast tumor tissues, lung metastatic tissues, and spleens from three 4T1 tumor-bearing BALB/c mice. Figure S5: the distribution of usage of TRBJ gene of CD4^+^CD25^+^ T cells in breast tumor tissues, lung metastatic tissues, and spleens from three 4T1 tumor-bearing BALB/c mice. Figure S6: the statistical analysis of usage of TRBV and TRBJ gene of CD4^+^CD25^+^ T cells in breast tumor tissues, lung metastatic tissues, and spleens from three 4T1 tumor-bearing BALB/c mice. Figure S7-1: the pairing usage of TRBV-TRBJ gene of CD4^+^CD25^+^ T cells in breast tumor tissues, lung metastatic tissues, and spleens from 4T1 tumor-bearing BALB/c mouse 1. Figure S7-2: the pairing usage of TRBV-TRBJ gene of CD4^+^CD25^+^ T cells in breast tumor tissues, lung metastatic tissues, and spleens from 4T1 tumor-bearing BALB/c mouse 2. Figure S7-3: the pairing usage of TRBV-TRBJ gene of CD4^+^CD25^+^ T cells in breast tumor tissues, lung metastatic tissues, and spleens from 4T1 tumor-bearing BALB/c mouse 3. Figure S8: the length distribution of CDR3 AA of CD4^+^CD25^+^ T cells in breast tumor tissues, lung metastatic tissues, and spleens from three 4T1 tumor-bearing BALB/c mice. Figure S9: the usage distribution of CDR3 AA of CD4^+^CD25^+^ T cells in breast tumor tissues, lung metastatic tissues, and spleens from three 4T1 tumor-bearing BALB/c mice. Supplement Table 1-1: DNA concentration and purity (Adaptive Biotechnologies Immuno SEQ completed) of CD4^+^CD25^+^ T cells in breast tumor tissues, lung metastatic tissues, and spleens from three 4T1 tumor-bearing BALB/c mice. Supplement Table 1-2: DNA concentration and purity (Adaptive Biotechnologies Immuno SEQ completed) of CD4^+^CD25^+^ T cells in breast tumor tissues, lung metastatic tissues, and spleens from three 4T1 tumor-bearing BALB/c mice. Supplement Table 2: the percentage of total CDR3 sequences, productive CDR3 sequences, unique CDR3 sequences, and unique productivity of CD4^+^CD25^+^ T cells TCR *β* CDR3 repertoire in breast tumor tissues, lung metastatic tissues, and spleens from three 4T1 tumor-bearing BALB/c mic. Supplement Table 3: the original sequencing results of the nine samples of CDR3 are given in the database (website) names and web pages (link addresses) published by Adaptive Biotechnologies Immuno SEQ company. ImmunoSEQ ANALYZER https://clients.adaptivebiotech.com/login?message=logout (ZT20160219B4, ZT20160219L5, ZT20160219SP6, ZT20160222B10, ZT20160222L11, ZT20160222SP12, ZT20160225B16, ZT20160225L17, and ZT20160225SP18). Supplement Table 4-1: the number of total public (or overlap) CDR3 sequences of CD4^+^CD25^+^ T cells TCR *β* unique CDR3 repertoire in three tissues from each 4T1 tumor-bearing BALB/c mice; and the number and ratio of public CDR3 sequences from their respective productive CDR3 repertoire in each tissues. Supplement Table 4-2: the number of total public (or overlap) CDR3 sequences of CD4^+^CD25^+^ T cells TCR *β* unique CDR3 repertoire in same tissues from three 4T1 tumor-bearing BALB/c mice, and the ratio of the number of public CDR3 sequences from their respective productive CDR3 repertoire between each two mice.
