# Supplementary material for: Analysis of the Heterogeneity of CD4+CD25+ T Cell TCR β CDR3 Repertoires in Breast Tumor Tissues, Lung Metastatic Tissues, and Spleens from 4T1 Tumor-Bearing BALB/c Mice
Source: J Immunol Res. 2020 Sep 24;2020:3184190. doi: 10.1155/2020/3184190 (PMC7532420; doi:10.1155/2020/3184190)
Supplement: Supplementary Materials — Figure S1: total DNA 1% agarose gel electrophoresis and DNA concentration. Figure S2: the mean clone percentage and statistical analysis of high-frequency (>0.05%), intermediate-frequency (0.01–0.05%), and low frequency (<0.01%) CD4+CD25+ T cells TCR β CDR3 AA repertoire in three same tissues from three 4T1 tumor-bearing BALB/c mice. Figure S3: the ratio of cloning proliferation of total overlap eight CDR3 sequences from CDR3 repertoire in each tissues among breast tumor tissues, lung metastatic tissues, and spleens from three 4T1 tumor-bearing BALB/c mice (total of nine tissues). Figure S4: the distribution of the usage of TRBV gene of CD4+CD25+ T cells in breast tumor tissues, lung metastatic tissues, and spleens from three 4T1 tumor-bearing BALB/c mice. Figure S5: the distribution of usage of TRBJ gene of CD4+CD25+ T cells in breast tumor tissues, lung metastatic tissues, and spleens from three 4T1 tumor-bearing BALB/c mice. Figure S6: the statistical analysis of usage of TRBV and TRBJ gene of CD4+CD25+ T cells in breast tumor tissues, lung metastatic tissues, and spleens from three 4T1 tumor-bearing BALB/c mice. Figure S7-1: the pairing usage of TRBV-TRBJ gene of CD4+CD25+ T cells in breast tumor tissues, lung metastatic tissues, and spleens from 4T1 tumor-bearing BALB/c mouse 1. Figure S7-2: the pairing usage of TRBV-TRBJ gene of CD4+CD25+ T cells in breast tumor tissues, lung metastatic tissues, and spleens from 4T1 tumor-bearing BALB/c mouse 2. Figure S7-3: the pairing usage of TRBV-TRBJ gene of CD4+CD25+ T cells in breast tumor tissues, lung metastatic tissues, and spleens from 4T1 tumor-bearing BALB/c mouse 3. Figure S8: the length distribution of CDR3 AA of CD4+CD25+ T cells in breast tumor tissues, lung metastatic tissues, and spleens from three 4T1 tumor-bearing BALB/c mice. Figure S9: the usage distribution of CDR3 AA of CD4+CD25+ T cells in breast tumor tissues, lung metastatic tissues, and spleens from three 4T1 tumor-bearing BALB/c mice. Supplement T [file 3184190.f1.docx]

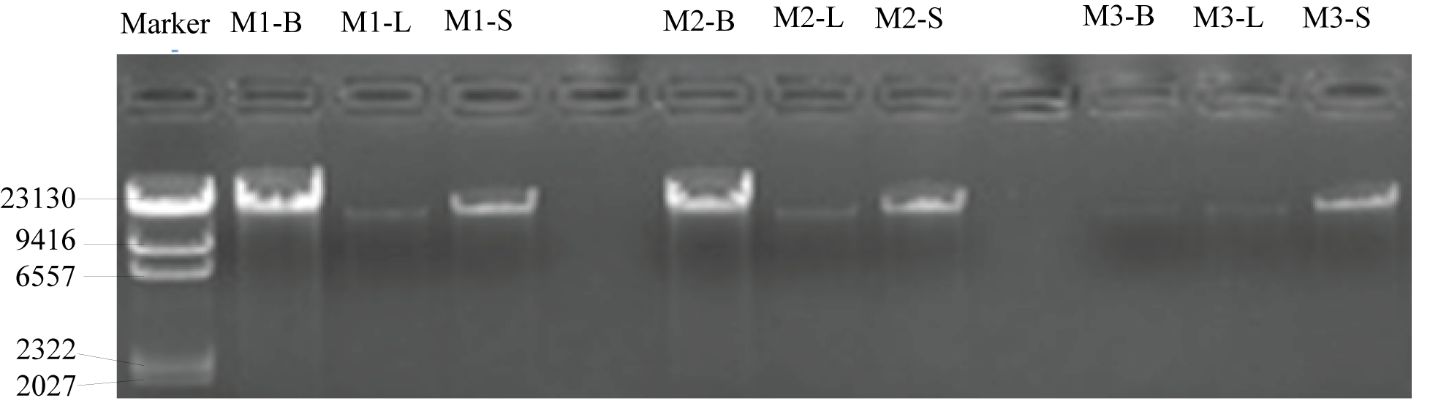


Figure S1. Total DNA 1% agarose gel electrophoresis and DNA concentration.


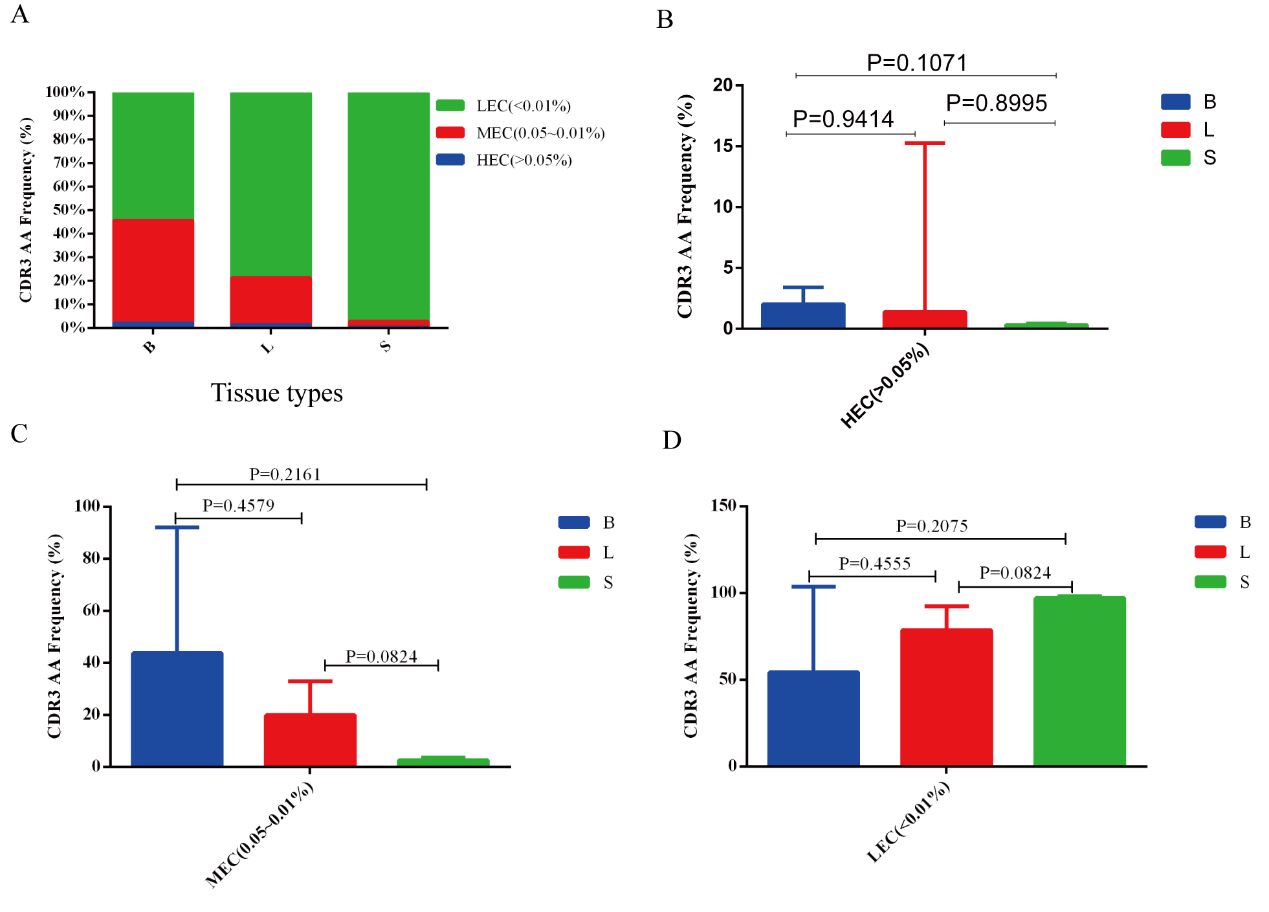


Figure S2. The mean clones percentage and statistical analysis of high-frequency (> 0.05%), intermediate-frequency (0.01–0.05%), and low frequency (<0.01%) of CD4^+^CD25^+^ T cells TCR β CDR3 AA repertoire in three same tissues from three 4T1 tumor-bearing BALB / c mice.


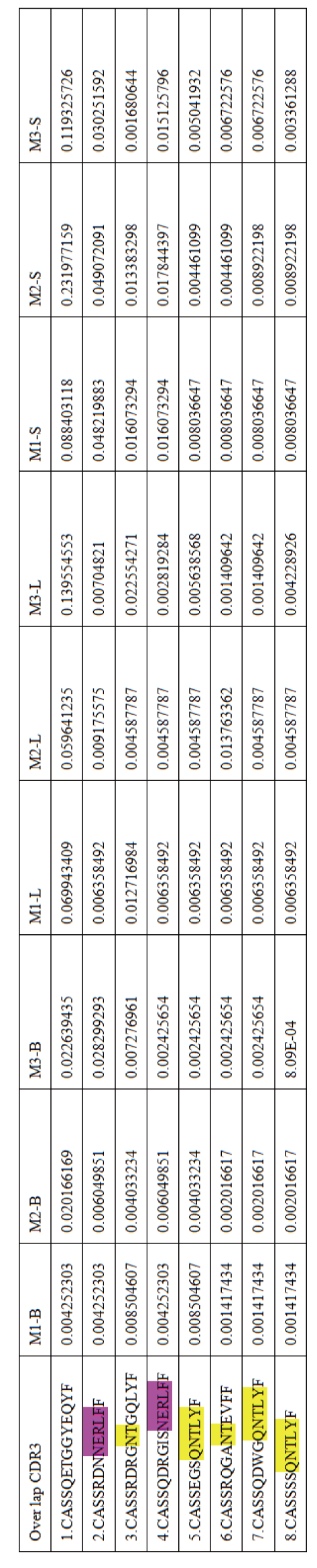


Figure S3. The ratio of cloning proliferation of total overlap eight CDR3 sequences from CDR3 repertoire in each tissues among breast tumor tissues, lung metastatic tissues, spleens from three 4T1 tumor-bearing BALB/c mice (total of nine tissues)


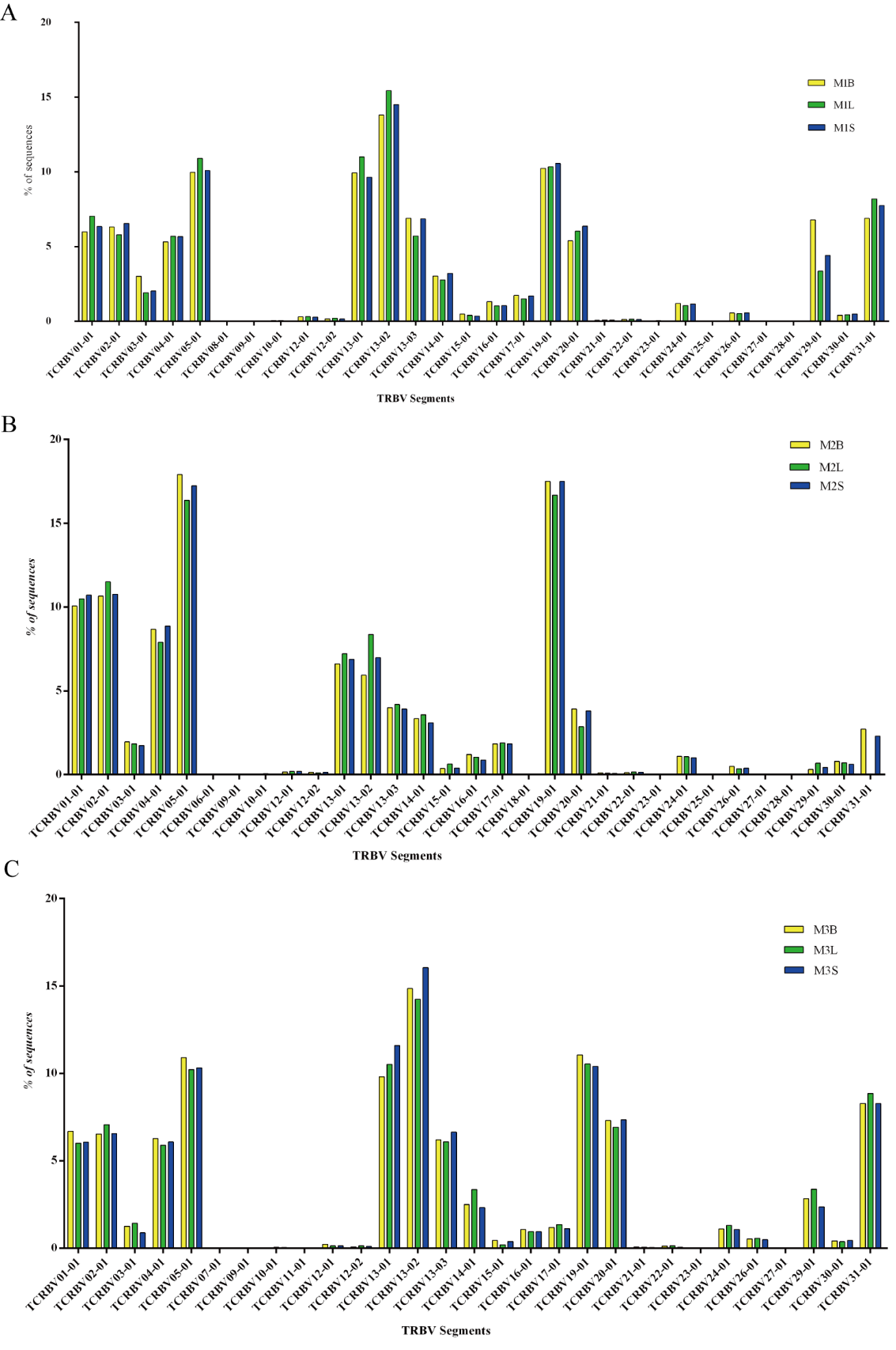


Figure S4. The distribution of usage of TRBV gene of CD4^+^CD25^+^ T cells in breast tumor tissues, lung metastatic tissues, spleens from three 4T1 tumor-bearing BALB/c mice.


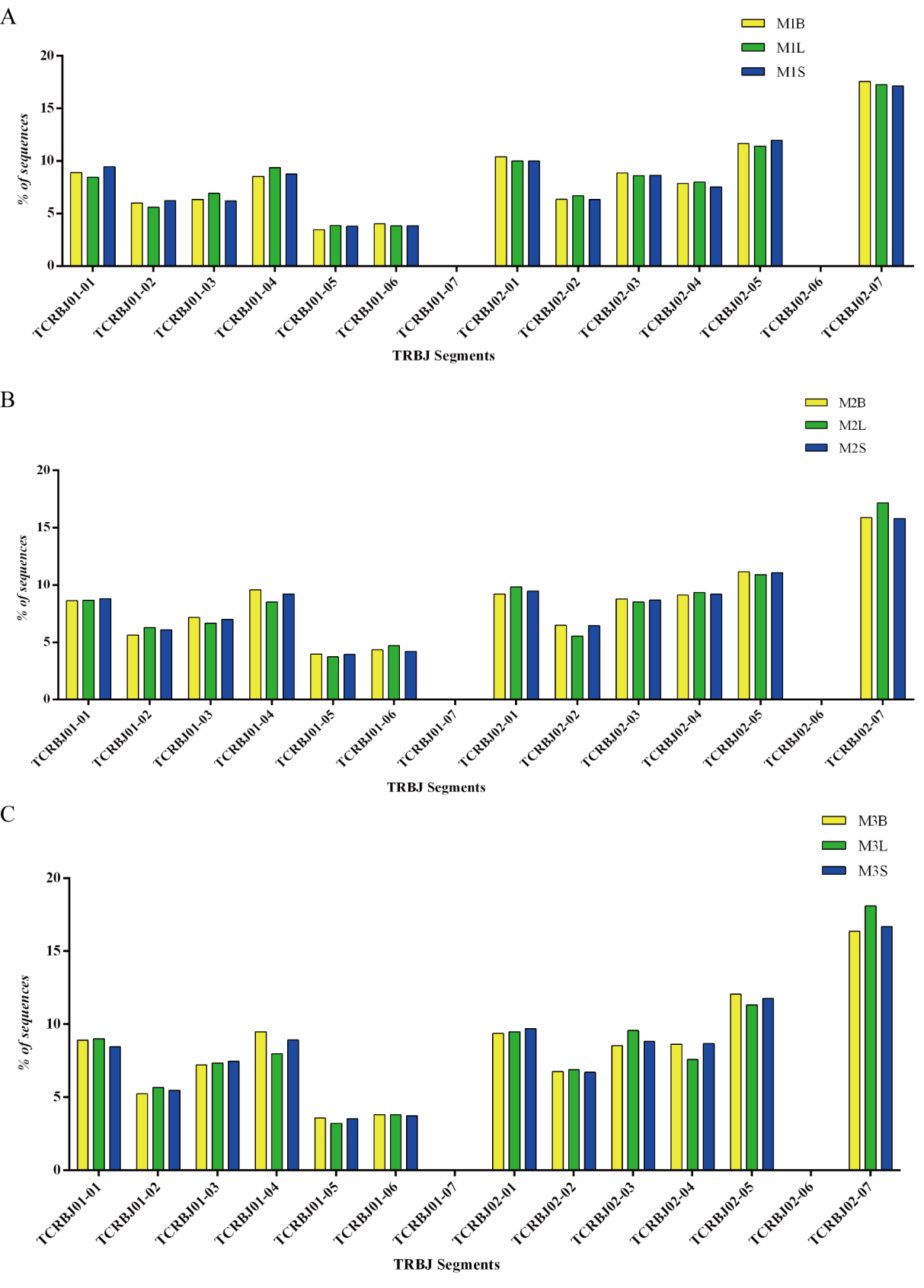


Figure S5. The distribution of usage of TRBJ gene of CD4^+^CD25^+^ T cells in breast tumor tissues, lung metastatic tissues, spleens from three 4T1 tumor-bearing BALB/c mice.


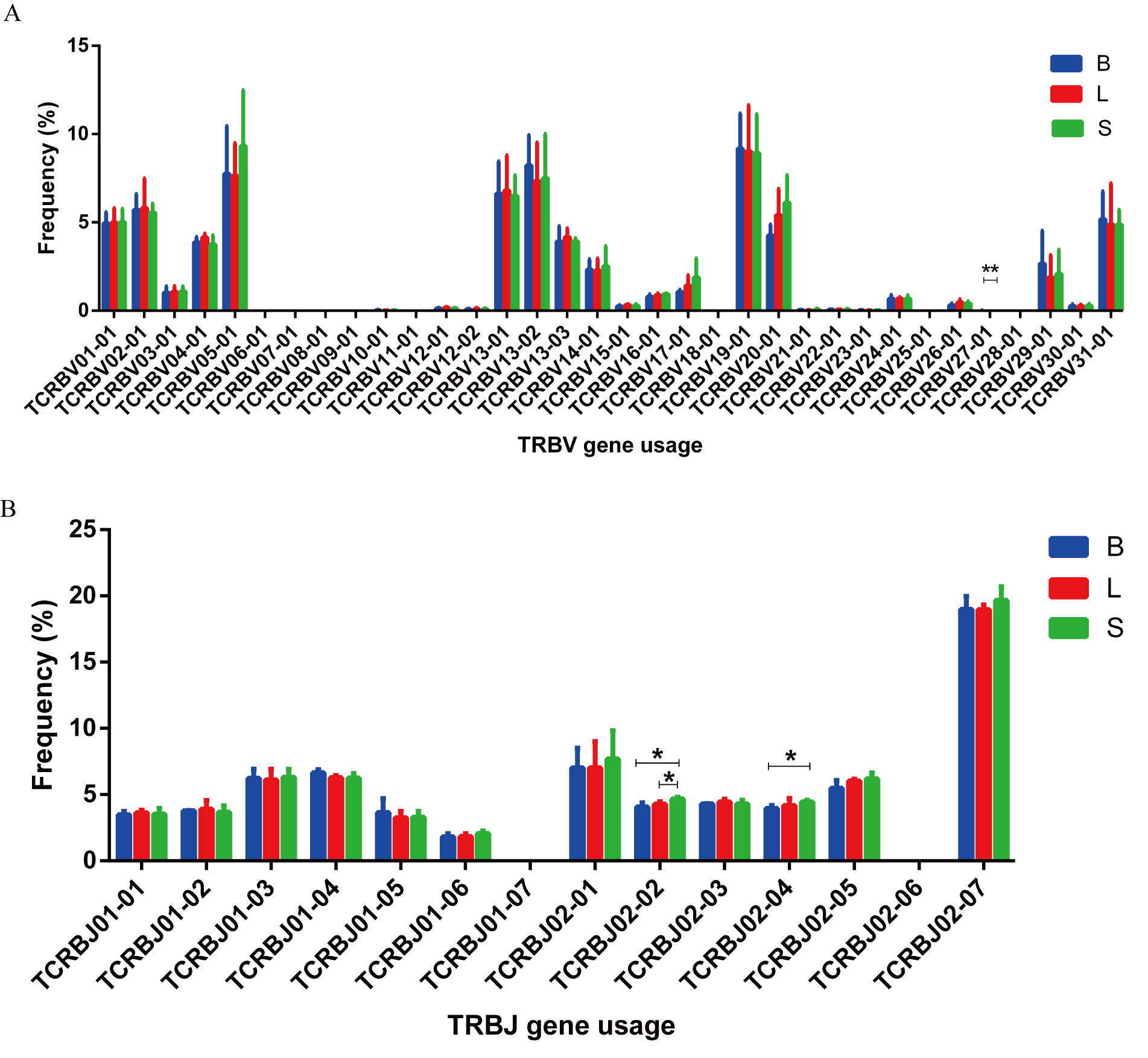


Figure S6. The statistical analysis of usage of TRBV&TRBJ gene of CD4^+^ CD25^+^ T cells in breast tumor tissues, lung metastatic tissues, spleens from three 4T1 tumor-bearing BALB/c mice.


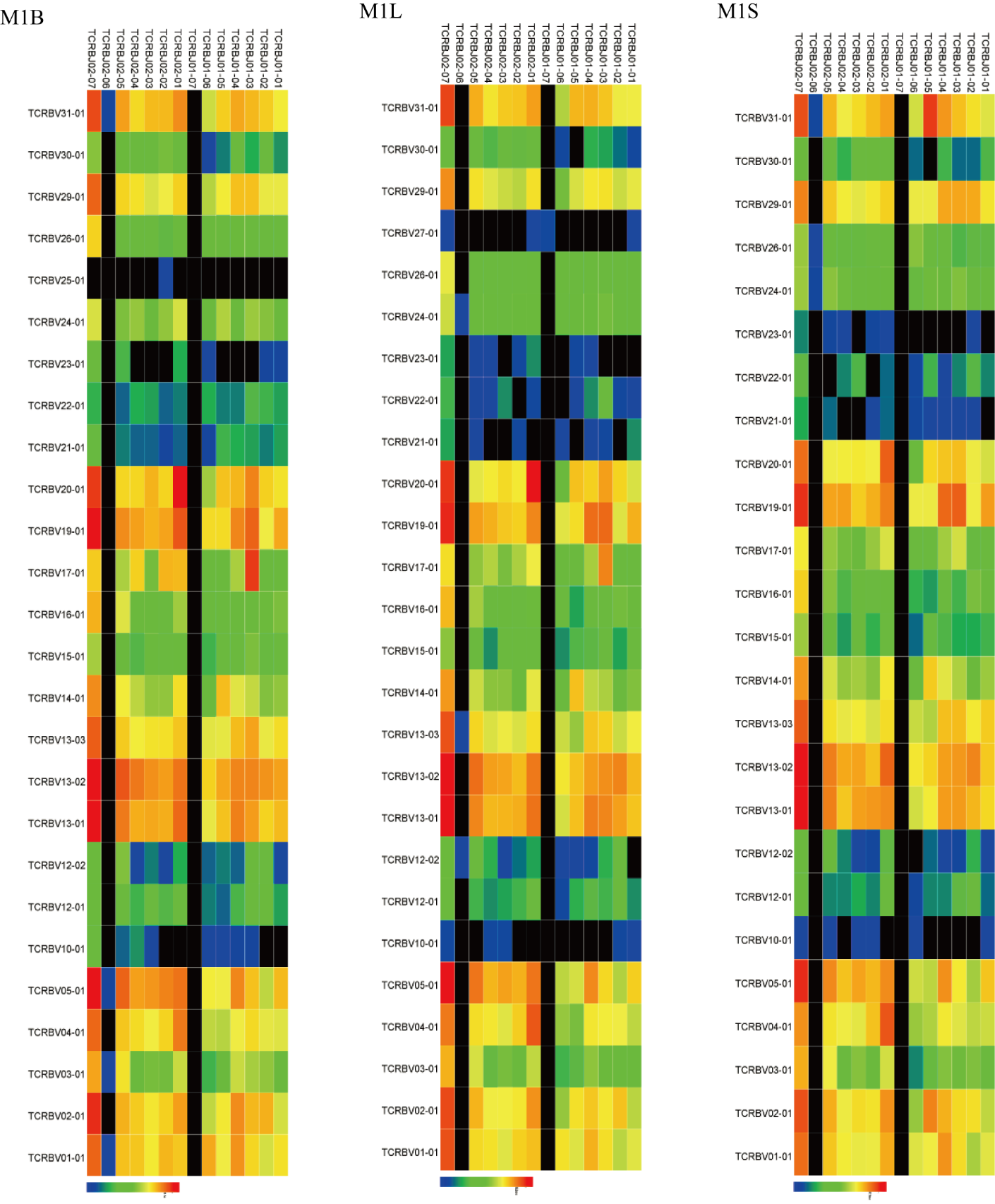


Figure S7-1. The pairing usage of TRBV-TRBJ gene of CD4^+^CD25^+^ T cells in breast tumor tissues, lung metastatic tissues, spleens from 4T1 tumor-bearing BALB/c mouse 1.


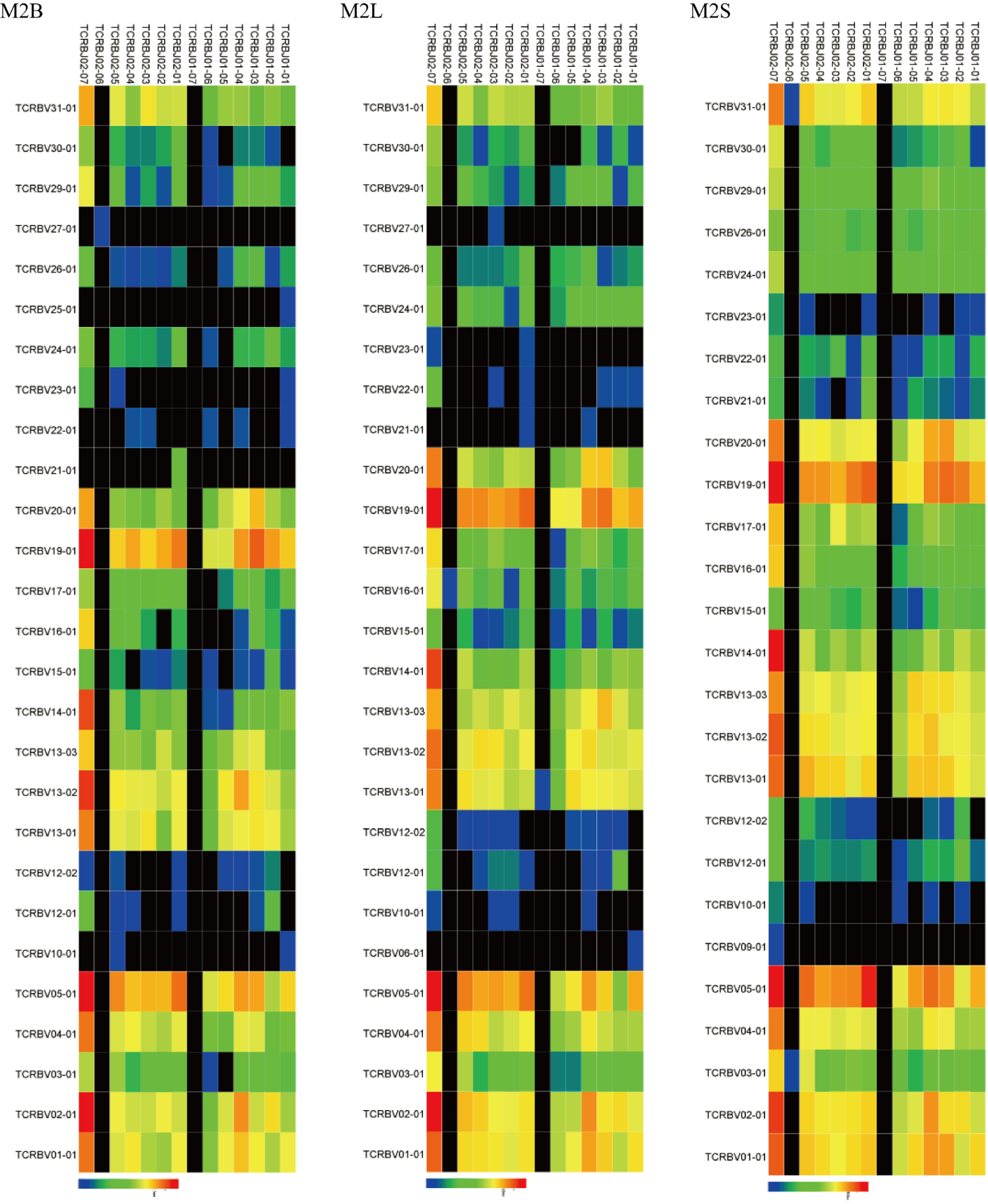


Figure S7-2. The pairing usage of TRBV-TRBJ gene of CD4^+^CD25^+^ T cells in breast tumor tissues, lung metastatic tissues, spleens from 4T1 tumor-bearing BALB/c mouse 2.


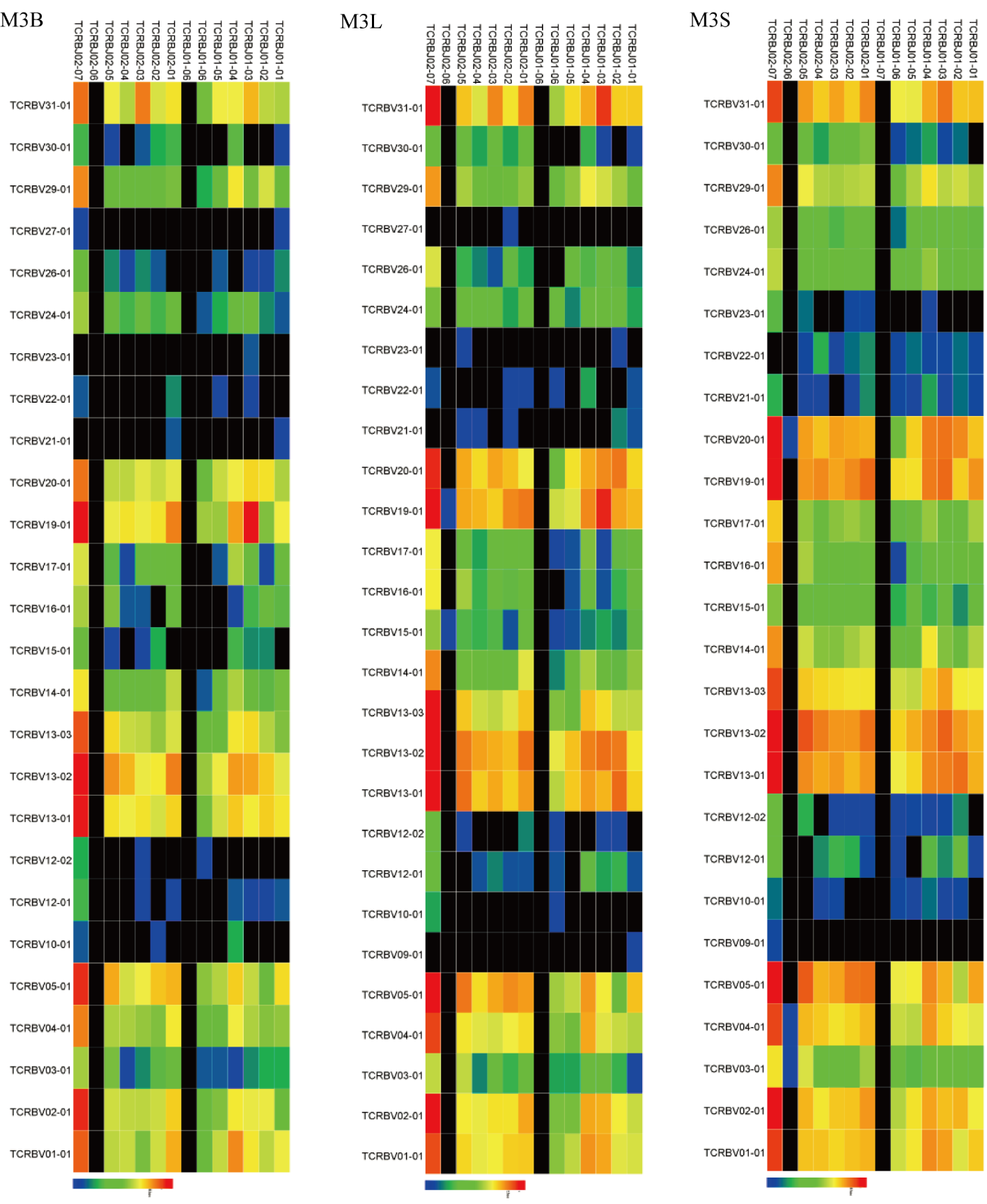


Figure S7-3. The pairing usage of TRBV-TRBJ gene of CD4^+^CD25^+^ T cells in breast tumor tissues, lung metastatic tissues, spleens from 4T1 tumor-bearing BALB/c mouse 3.


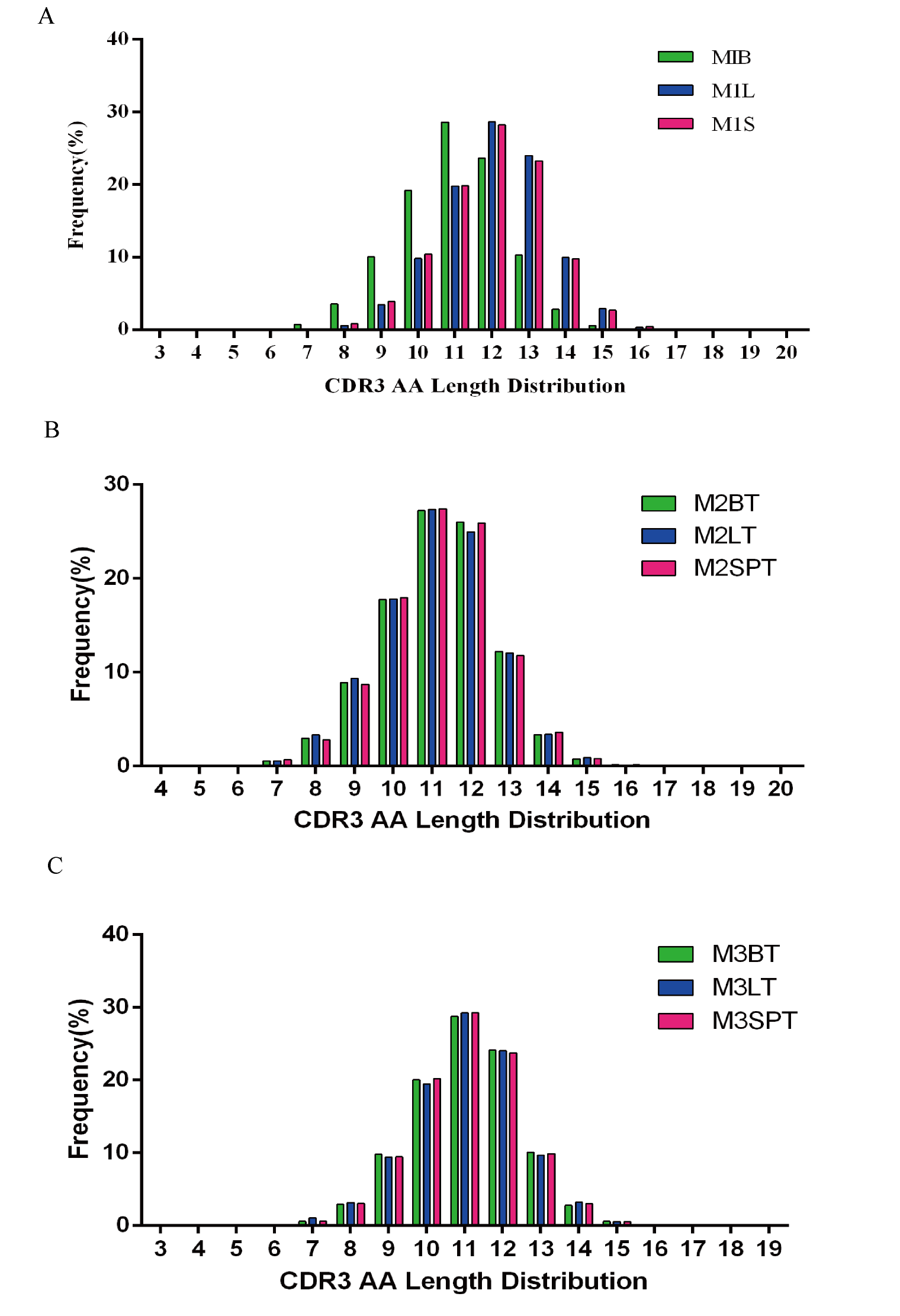


Figure S8. The length distribution of CDR3 AA of CD4^+^CD25^+^ T cells in breast tumor tissues, lung metastatic tissues, spleens from three 4T1 tumor-bearing BALB/c mice.


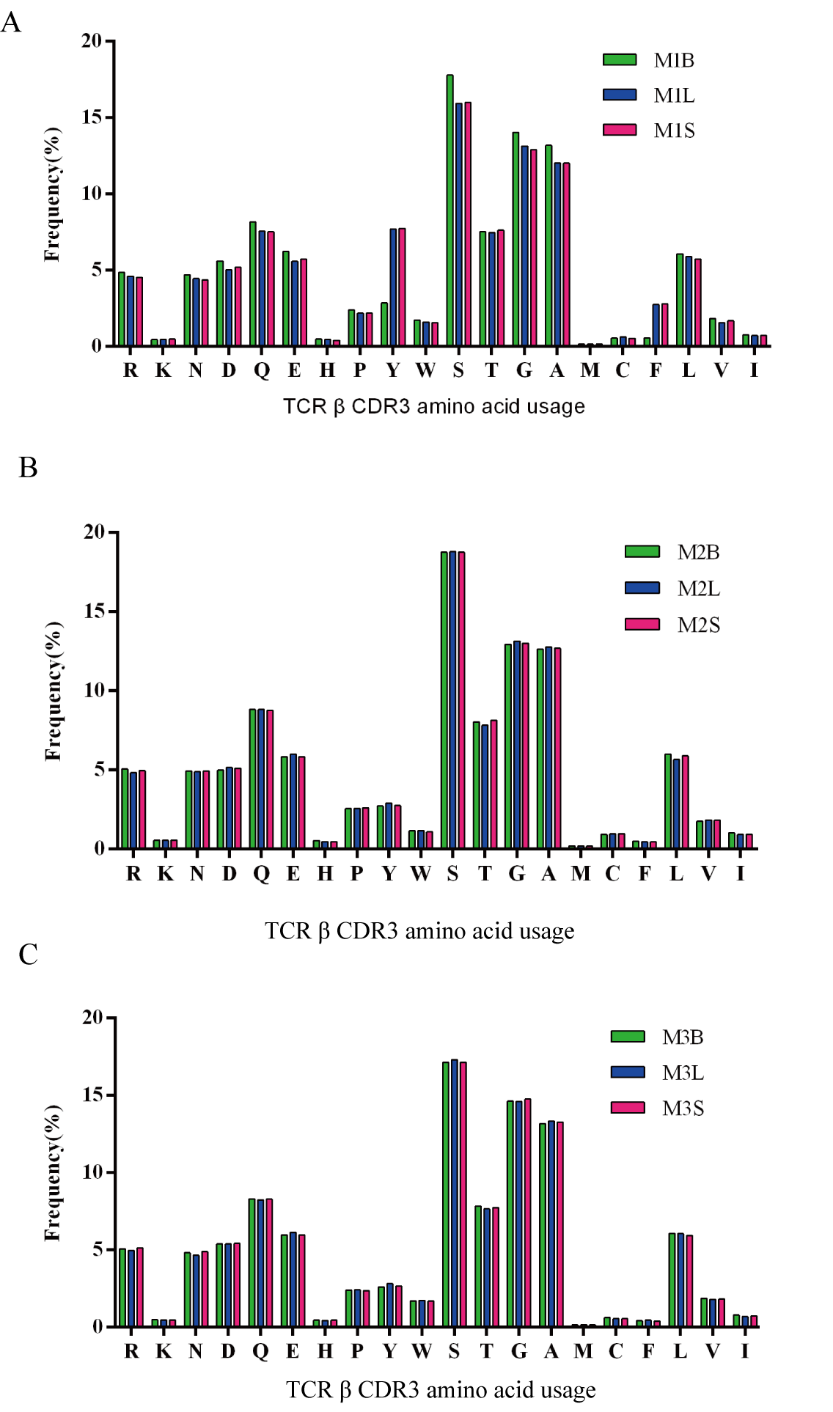


Figure S9. The usage distribution of CDR3 AA of CD4^+^CD25^+^ T cells in breast tumor tissues, lung metastatic tissues, spleens from three 4T1 tumor-bearing BALB/c mice.

**Supplement Table 1-1．**DNA concentration and purity (Adaptive Biotechnologies Immuno SEQ completed) of CD4^+^CD25^+^ T cells in breast tumor tissues, lung metastatic tissues, spleens from three 4T1 tumor-bearing BALB/c mice.

| Pass/Fail | Sample ID | Average Conc.(ng/μl) | Conc. Variation | Passing Standards |
| --- | --- | --- | --- | --- |
| Pass | 0 Drop Quant Controls TE | 0.96 | 0.5625 | Avg. Conc. -5 to 5ng/μl |
| Pass | 10 Drop Quant Controls Calf Thymus 150511 TE | 11.03 | 0.0018 | Avg. Conc. 5 to 15ng/μl : <20% variation |
| Pass | 200 Drop Quant Controls Calf Thymus 150511 TE | 192.945 | 0.0008 | Avg. Conc. 180 to 220ng/μl : <20% variation |

**Supplement Table 1-2．**DNA concentration and purity (Adaptive Biotechnologies Immuno SEQ completed) of CD4^+^CD25^+^ T cells in breast tumor tissues, lung metastatic tissues, spleens from three 4T1 tumor-bearing BALB/c mice.

| Sample ID | Type | Species | Locus | Resolution | Source | Conc.(ng/μl) 1 | Conc.(ng/μl) 2 | Average Conc.(ng/μl) | Conc. Variation | Average |
| --- | --- | --- | --- | --- | --- | --- | --- | --- | --- | --- |
| ZT2016021 | gDNA | Mouse | TCRB | Survey | PBMC | 113.68 | 112.33 | 113.005 | 0.0119 | 1.785 |
| ZT2016021 | gDNA | Mouse | TCRB | Survey | PBMC | 159.89 | 161.57 | 160.73 | 0.0105 | 1.775 |
| ZT2016021 | gDNA | Mouse | TCRB | Survey | PBMC | 137.46 | 136.35 | 136.905 | 0.0081 | 1.82 |
| ZT2016021 | gDNA | Mouse | TCRB | Survey | PBMC | 47.69 | 48.92 | 48.305 | 0.0255 | 1.56 |
| ZT2016021 | gDNA | Mouse | TCRB | Survey | PBMC | 46.78 | 46.97 | 46.875 | 0.0041 | 1.49 |
| ZT2016021 | gDNA | Mouse | TCRB | Survey | PBMC | 108.37 | 102.94 | 105.655 | 0.0514 | 1.7 |
| ZT2016022 | gDNA | Mouse | TCRB | Survey | PBMC | 40.25 | 39.54 | 39.895 | 0.0178 | 1.595 |
| ZT2016022 | gDNA | Mouse | TCRB | Survey | PBMC | 198.89 | 202.23 | 200.56 | 0.0167 | 1.75 |
| ZT2016022 | gDNA | Mouse | TCRB | Survey | PBMC | 226.55 | 226.39 | 226.47 | 0.0007 | 1.83 |

**Supplement Table 2.** The percentage of total CDR3 sequences, productive CDR3 sequences, unique CDR3 sequences and unique from productive of CD4^+^CD25^+^ T cells TCR β CDR3 repertoire in breast tumor tissues, lung metastatic tissues, spleens from three 4T1 tumor-bearing BALB/c mice.

| Sample Name | Total CDR3 Sequences | Total Productive CDR3 Sequences | Unique Productive CDR3 Sequences | Unique  /Total (%) |
| --- | --- | --- | --- | --- |
| M1-B | **70550** | **51021** | **25978** | **50.92%** |
| M2-B | 15727 | 10668 | 5994 | 56.19% |
| M3-B | 12443 | 8528 | 5112 | 59.94% |
| M1-L | 49588 | 35626 | 19214 | 53.93% |
| M2-L | 21797 | 14840 | 9494 | 63.98% |
| M3-L | 22416 | 15736 | 8459 | 53.76% |
| M1-S | 123678 | 91435 | 44993 | 49.21% |
| M2-S | 70940 | 50590 | 25199 | 49.81% |
| M3-S | 59501 | 42550 | 26676 | 62.69% |

**Supplement Table 3**. The original sequencing results of the nine samples CDR3 are given in the database (website) names and web pages (link addresses) published by Adaptive Biotechnologies Immuno SEQ company.

ImmunoSEQ ANALYZER

https://clients.adaptivebiotech.com/login?message=logout

[ZT20160219B4, ZT20160219L5, ZT20160219SP6, ZT20160222B10, ZT20160222L11, ZT20160222SP12, ZT20160225B16, ZT20160225L17, ZT20160225SP18. ]

| Sample Name | Total | Unique | Productive Total | Productive Unique | Out of Frame Total | Out of Frame Unique | Has Stop Total | Has Stop Unique | Entropy | Clonality | Mass of Reaction Template (ng) | Max Frequency (%) | Gene Rearrangements |
| --- | --- | --- | --- | --- | --- | --- | --- | --- | --- | --- | --- | --- | --- |
| M1B | 70550 | 36725 | 51021 | 25978 | 18885 | 10342 | 644 | 405 | 13.422196 | 0.08474644 | 1212.16 | 1.339475549 | 70550 |
| M1L | 49588 | 28075 | 35626 | 19214 | 13531 | 8575 | 431 | 286 | 13.312524 | 0.06446623 | 1212.16 | 1.712107768 | 49588 |
| M1S | 123678 | 63463 | 91435 | 44993 | 31211 | 17877 | 1032 | 593 | 13.883521 | 0.101821154 | 1212.16 | 2.590598166 | 123678 |
| M2B | 15727 | 9163 | 10668 | 5994 | 4915 | 3084 | 144 | 85 | 11.956688 | 0.04722301 | 772.48 | 0.86475488 | 15727 |
| M2L | 21797 | 14317 | 14840 | 9494 | 6763 | 4673 | 194 | 150 | 12.709728 | 0.03807459 | 1212.16 | 0.9037941 | 21797 |
| M2S | 70940 | 36243 | 50590 | 25199 | 19804 | 10705 | 546 | 339 | 13.398437 | 0.08362191 | 632.64 | 1.946715534 | 70940 |
| M3B | 12443 | 7911 | 8528 | 5112 | 3794 | 2717 | 121 | 82 | 11.823585 | 0.04026791 | 1212.16 | 1.293900185 | 12443 |
| M3L | 22416 | 12664 | 15736 | 8459 | 6486 | 4080 | 194 | 125 | 12.286507 | 0.058236144 | 962.24 | 0.539793005 | 22416 |
| M3S | 59501 | 38438 | 42550 | 26676 | 16411 | 11393 | 540 | 369 | 14.101414 | 0.040932525 | 1212.16 | 0.608393136 | 59501 |

**Supplement Table 4-1.** The number of total public (or overlap) CDR3 sequences of CD4^+^CD25^+^ T cells TCR β unique CDR3 repertoire in three tissues from each 4T1 tumor-bearing BALB/c mice; and the number and ratio of public CDR3 sequences from their respective productive CDR3 repertoire in each tissues.

| **Sample** | **M1-B** | **M1-L** | **M1-S** |
| --- | --- | --- | --- |
| **M1-B** | 23314 | 3939(22.60%) | 5972(15.13%) |
| **M1-L** | 3939(16.90%) | 17431 | 4958(12.56%) |
| **M1-S** | 5972(25.62%) | 4958(28.44%) | 39462 |
|  | **M2-B** | **M2-L** | **M2-S** |
| **M2-B** | 5635 | 1070(11.97%) | 1873(8.20%) |
| **M2-L** | 1070(18.99%) | 8937 | 2496(10.92%) |
| **M2-S** | 1873(33.24%) | 2496(27.93%) | 22853 |
|  | **M3-B** | **M3-L** | **M3-S** |
| **M3-B** | 9380 | 734(9.42%) | 1244(15.16%) |
| **M3-L** | 734(7.83%) | 7795 | 414(5.05%) |
| **M3-S** | 1538(16.40%) | 2195（28.16%) | 8206 |

**Supplement Table 4-2.** The number of total public (or overlap) CDR3 sequences of CD4^+^CD25^+^ T cells TCR β unique CDR3 repertoire in same tissues from three 4T1 tumor-bearing BALB/c mice, and the ratio of the number of public CDR3 sequences from their respective productive CDR3 repertoire between each two mice.

| **Sample** | **M1-B** | **M2-B** | **M3-B** |
| --- | --- | --- | --- |
| **M1-B** | 23314 | 551(9.78%) | 862(9.19%) |
| **M2-B** | 551(2.36%) | 5635 | 229(2.44%) |
| **M3-B** | 862(3.70%) | 229（4.06%) | 9380 |
|  | **M1-L** | **M2-L** | **M3-L** |
| **M1-L** | 17431 | 665(7.44%) | 569(7.30%) |
| **M2-L** | 665(3.82%) | 8937 | 337(4.32%) |
| **M3-L** | 569(3.26%) | 337（3.77%） | 7795 |
|  | **M1-S** | **M2-S** | **M3-S** |
| **M1-S** | 39462 | 2914(12.75%) | 3110(12.80%) |
| **M2-S** | 2914(7.38%) | 22853 | 2037(8.39%) |
| **M3-S** | 3110(7.88%) | 2037（8.91%) | 24288 |
